# Dormant 5-lipoxygenase in inflammatory macrophages is triggered by exogenous arachidonic acid

**DOI:** 10.1038/s41598-017-11496-3

**Published:** 2017-09-08

**Authors:** Carlos A. Sorgi, Simona Zarini, Sarah A. Martin, Raphael L. Sanchez, Rodrigo F. Scandiuzzi, Miguel A. Gijón, Carlos Guijas, Nicolas Flamand, Robert C. Murphy, Lucia H. Faccioli

**Affiliations:** 1 Departamento de Análises Clínicas, Toxicológicas e Bromatológicas, Faculdade de Ciências Farmacêuticas de Ribeirão Preto, Universidade de São Paulo, Ribeirão Preto, SP 14040-903 Brazil; 2 Department of Pharmacology, University of Colorado Denver, Aurora, 80045 CO USA; 3 Scripps Center for Metabolomics, The Scripps Research Institute, La Jolla, 92037 CA USA; 4 Centre de Recherche de l’Institut Universitaire de Cardiologie et de Pneumologie de Québec, Département de Médecine, Faculté de Médecine, Université Laval, Quebec City, G1V 4G5 Quebec Canada

## Abstract

The differentiation of resident tissue macrophages from embryonic precursors and that of inflammatory macrophages from bone marrow cells leads to macrophage heterogeneity. Further plasticity is displayed through their ability to be polarized as subtypes M1 and M2 in a cell culture microenvironment. However, the detailed regulation of eicosanoid production and its involvement in macrophage biology remains unclear. Using a lipidomics approach, we demonstrated that eicosanoid production profiles between bone marrow-derived (BMDM) and peritoneal macrophages differed drastically. In polarized BMDMs, M1 and M2 phenotypes were distinguished by thromboxane B_2_, prostaglandin (PG) E_2_, and PGD_2_ production, in addition to lysophospholipid acyltransferase activity. Although *Alox5* expression and the presence of 5-lipoxygenase (5-LO) protein in BMDMs was observed, the absence of leukotrienes production reflected an impairment in 5-LO activity, which could be triggered by addition of exogenous arachidonic acid (AA). The BMDM 5-LO regulatory mechanism was not responsive to PGE_2_/cAMP pathway modulation; however, treatment to reduce glutathione peroxidase activity increased 5-LO metabolite production after AA stimulation. Understanding the relationship between the eicosanoids pathway and macrophage biology may offer novel strategies for macrophage-associated disease therapy.

## Introduction

Mononuclear phagocytes such as monocytes, macrophages, dendritic cells, and their bone marrow progenitors, play a major role in inflammation by eliminating pathogens and producing soluble mediators^1^. The manifold origins of macrophages are well described in mouse^2^, wherein it is postulated that the resident tissue macrophages (Res-MAs), such as from the peritoneum (PM), are not differentiated from blood monocytes but rather originate from an embryonic precursor and are maintained by self-renewal^3, 4^. However, under inflammatory conditions, infiltrating monocytes from bone marrow progenitors are also found that can differentiate into inflammatory macrophages (Infl-MAs)^5^. Accordingly, bone marrow-derived macrophages (BMDMs), which are derived from bone marrow cells cultured *in vitro* in the presence of growth factors, represent a useful system for the investigation of Infl-MA functions^6^. Subsequently, the phenotype of macrophages can be polarized by the microenvironment^7^. Essentially, macrophages can modify their functions between M1 macrophages (activated by interferon γ (IFN-γ), interleukin (IL)-1β, and lipopolysaccharide (LPS)) to produce inflammatory mediators; and M2 macrophages that mediate tissue repair injury and produce anti-inflammatory factors (activated by IL-4, IL-13, and the immune-complexes)^8^. Previous studies on macrophage systems to define such phenotypes and their roles in immunology has been based mostly on cytokine and chemokine profiles. Instead, the eicosanoid pattern has been demonstrated on macrophage subsets, detailed biochemistry pathway relating to eicosanoid production and function may also play specific roles within the larger context of macrophage biology.

Lipid mediators such as eicosanoids act as hormone-like factors in biological processes and display diverse functions in the immune system^9^. Arachidonic acid (AA) esterified at phospholipids serves as the substrate for activation-induced eicosanoid biosynthesis^10^, including prostaglandins (PGs), thromboxane (TX), leukotrienes (LTs), and lipoxins (LXs)^9, 11^. AA is released upon hydrolysis catalysed by phospholipases A_2_, notably the group IVA phospholipase A2 (cPLA_2_)^12^. Cyclooxygenases (COX-1 and COX-2) catalyse AA oxidation to prostanoids and specific PGs were formed after various synthase activity^10^. Furthermore, AA can be converted via another pathway to LTs by 5-lipoxygenase (5-LO)^13^. 5-LO translocates to the nuclear envelope upon cell activation and catalyses the formation of 5-hydroperoxyeicosatetraenoic acid (5-HpETE) and subsequently LTA_4_^13^. In turn, 5-HpETE can be reduced by a peroxidase to form 5- hydroxyeicosatetraenoic acid (5-HETE). LTA_4_ can be enzymatically hydrolysed by LTA_4_ hydrolase to generate LTB_4_, or conjugated with glutathione by LTC_4_ synthase to generate LTC_4_, and further metabolized to LTD_4_ and LTE_4_, collectively known as cysteinyl leukotrienes (cys-LTs). Particularly, in an aqueous environment LTA_4_ is quickly and non-enzymatically degraded to 6-*trans*-LTB_4_, 6-trans-12-epi-LTB4 or 5,6-diHETEs^14^. The 12/15-lipoxygenase (12-LO/15-LO) pathway of AA metabolism produces predominantly 12-HETE and 15-HETE^15^. Notably, the reacylation of AA into phospholipids (Lands’ cycle) through the concerted actions of cPLA_2_ and lysophospholipid acyltransferases (LPATs) represents an important metabolic point of control during eicosanoid production^16, 17^.

Toll-like receptor 4 (TLR4)-specific agonist KLA stimulated eicosanoids release in murine macrophages^18^. In different macrophage phenotypes (resident PM, thioglycollate-elicited peritoneal macrophages (TGEMs), BMDMs, and immortalized RAW264.7 (RAW) cells), eicosanoids production almost exclusively comprises COX pathway metabolites^19^. However, the data related to the 5-LO pathway in macrophage diversity were inconclusive, in particular with regard to comparison between Res-MAs and Infl-MAs. Compared with well-known features of macrophage metabolism such as glucose consumption and lactate release, fatty acids, vitamins, and iron metabolism, which are also dependent on macrophage polarization and tissue specificity^20, 21^, the detailed eicosanoid metabolic pathways engaged in macrophage phenotypes (M1 and M2) have remained poorly characterized. Therefore, in the current study, we utilize mass spectrometry and molecular biology approaches to expand upon the current understanding of eicosanoid metabolism between macrophages of different sources of origin and polarization. We characterized the differences in eicosanoid production between Res-MAs and Infl-MAs, to identify a regulatory mechanism that could manage the 5-LO activity, leading to the observed metabolite formation in Infl-MAs. We further examined whether macrophage biology could impact eicosanoid metabolism through the effects of different stimulus pathways on the regulation of 5-LO. An enhanced understanding regarding the role of eicosanoids in the immune system may offer novel opportunities for macrophage-associated disease therapy.

## Results

### Comparative eicosanoid production in primary macrophages demonstrated a lipoxygenase pathway deficiency in BMDMs

**Figure 1:**
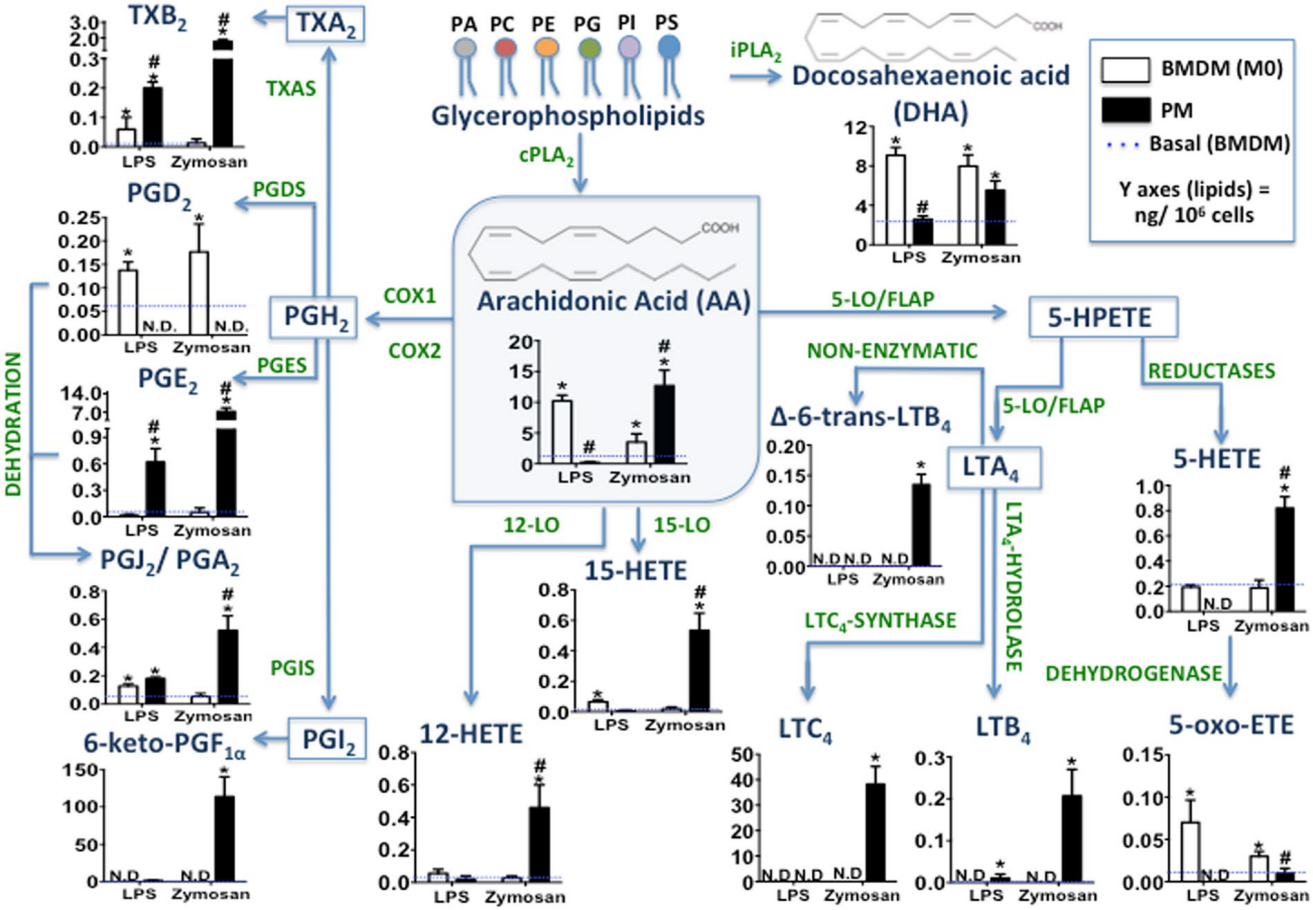
Comparative eicosanoid catabolism profile in BMDMs and PMs stimulated with microbial particles. BMDMs and PMs (1 × 10^6^ cells/well) were adhered on cell culture plates for 24 h. Macrophages were stimulated with LPS (500 ng/mL) or zymosan (30 particles/cell) for 6 and 1.5 h, respectively. The lipid mediators in cell culture supernatants were identified and quantified by HPLC-MS/MS (MRM mode) for eicosanoids: TXB_2_, PGD_2_, PGE_2_, PGJ_2_/PGA_2_, 6-keto-PGF_1α_, 12-HETE, 15-HETE, 5-HETE, 5-oxo-ETE, LTC_4_, LTB_4_, and Δ-6-*trans*-LTB_4_ as well as for the release of fatty acids: AA and DHA. Results are expressed as the means ± s.e.m. of three experiments (n = 3). Differences were considered when *p* < 0.05, *BMDMs and PMs stimulated or not, compared to non-stimulated basal BMDM production (dashed line), ^**#**^PM compared to BMDM eicosanoid production, after LPS or zymosan stimulation.

Understanding the complex networks of eicosanoid metabolism and signalling at the cellular level requires combinatory methodologies between analytical chemistry and molecular biology. Hence, we provided a lipidomic quantitative dataset of eicosanoids by HPLC-MS/MS and defined the profiles according to macrophage source of origin. Figure 1 shows a schematic of eicosanoid pathways. The membrane phospholipids comprise a major source of AA and DHA, and the release of both fatty acids was identified in macrophages. However, following LPS or zymosan stimulation, BMDMs released significantly more AA and DHA than did PMs. In the COX pathway (COX-1/COX-2), PGD_2_ appeared to be produced only in BMDMs, unlike other prostanoids analysed in PMs. Notably, when we used the EIA Kit method to quantify PGD_2_ and PGE_2_ in BMDMs stimulated with zymosan, the prostanoids were not distinguished as well using a MS approach (Supplementary Fig. S1). In the lipoxygenase pathway, we demonstrated higher production of LTs in PMs stimulated with zymosan. Additionally, PMs produced higher 5-HETE amounts after zymosan stimulation. However, the BMDM production of 5-LO metabolites was absent under these conditions. 5-HETE production was demonstrated in BMDMs, although at the same level as in non-stimulated macrophages. Conversely, 5-oxo-ETE production was higher in BMDMs than PMs, especially following stimulation with LPS. Furthermore, 12/15-LO metabolites production was identified in PMs stimulated by zymosan but not in BMDMs. Together, our results of comparison between BMDMs and PMs stimulated *in vitro* with the same microbial particles (zymosan or LPS), demonstrate a significant bias in eicosanoids production between these macrophage types.

### Eicosanoid profiling in BMDMs polarized by classical or alternative pathways post-stimulated with LPS or zymosan *in vitro*

**Figure 2:**
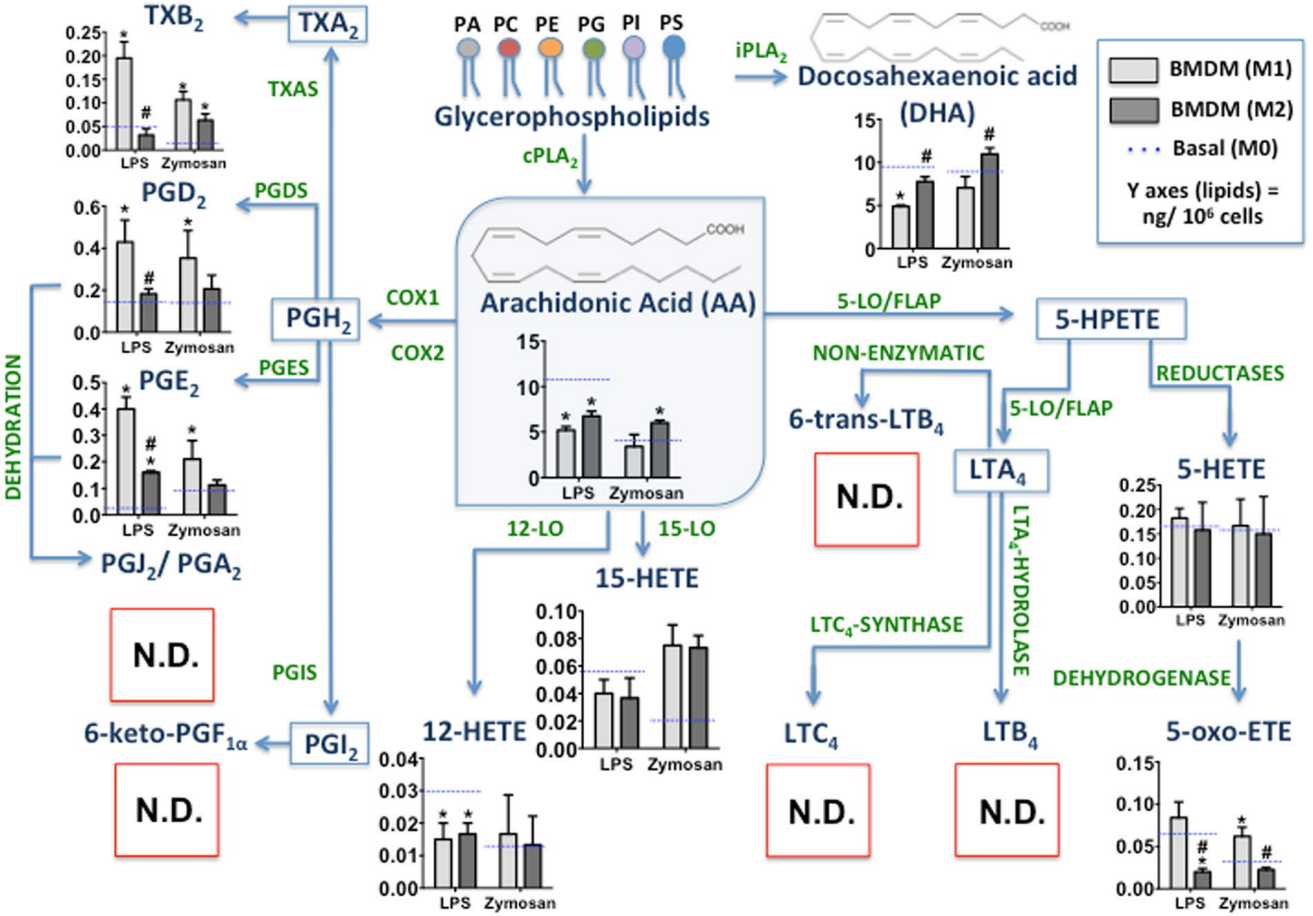
Effect of BMDM polarization on eicosanoids production after microbial particle stimulation. BMDMs were treated with IFN-γ (100 ng/mL) (M1), IL-4 + IL-13 (10 ng/mL) (M2), or only adhered (M0). Then, BMDMs were stimulated with LPS (500 ng/mL) or zymosan (30 particles/cell) for 6 and 1.5 h, respectively. The lipid mediators in cell culture supernatants were identified and quantified by HPLC-MS/MS (MRM mode) for eicosanoids: TXB_2_, PGD_2_, PGE_2_, PGJ_2_/PGA_2_, 6-keto-PGF_1α_, 12-HETE, 15-HETE, 5-HETE, 5-oxo-ETE, LTC_4_, LTB_4_, and Δ-6-*trans*-LTB_4_, as well as for the release of fatty acids: AA and DHA. The absence of specific eicosanoid production is represented by a red square (N.D.). Results are expressed as the means ± s.e.m. of three experiments (n = 3). Differences were considered significant when *p* < 0.05, *M1 and M2 compared to M0 and ^#^M2 compare to M1.

Firstly, we confirmed the protocols for BMDM polarization using different parameters as described in Supplementary Note. As shown in Fig. 2, the eicosanoids production by M1 and M2 compared to M0 differed in quantitative production, but not with respect to class of eicosanoids. The release of fatty acids such as AA and DHA differed between macrophage subtypes. For the cyclooxygenase pathway, we demonstrated increased production of TXB_2_, PGE_2_, and PGD_2_ in M1 compared to M2, especially following LPS stimulation. For the lipoxygenase pathway, we did not observe production of 5-LO-derivative metabolites in any conditions of BMDM polarization, regardless of the stimulus. Additionally, we observed non-statistically significant differences in the production of 5-HETE, 12-HETE, and 15-HETE between M1 and M2 for both stimuli. However, M1 production of 5-oxo-ETE was higher than that in M2. These results suggest that the polarization process of BMDMs did not modulate 5-LO activity.

GM-CSF priming of BMDMs enhanced the capacity to drive the pro-inflammatory response by TNF-α and NO production^22^. Additionally, this altered lipid mediator release and increased *Alox5* (5-LO) mRNA expression^23^. Thus, we primed BMDMs with IFN-γ and GM-CSF to obtain an M1 phenotype, which had the additional effect on increasing 5-LO expression. After priming, we stimulated BMDMs with A23187, a non-specific stimulus, and analysed the eicosanoids production. We observed increased production of TXB_2_, PGD_2_, and PGE_2_ after stimulation (Supplementary Fig. S3A), indicating the direct capacity of these macrophages to activate prostanoid catabolism. With respect to the lipoxygenase pathway, we demonstrated increased production of 5-HETE, 5-oxo-ETE, 12-HETE, and 15-HETE in GM-CSF-primed BMDMs after stimulation. However, we did not observe production of LTB_4_, Cys-Lts, or other LT-derived metabolites after priming with GM-CSF and A23187 post-stimulation.

### Transcript analysis of eicosanoid metabolism enzymes demonstrated the presence of 5-LO pathway components and the expression of the 5-LO protein in BMDMs

**Figure 3:**
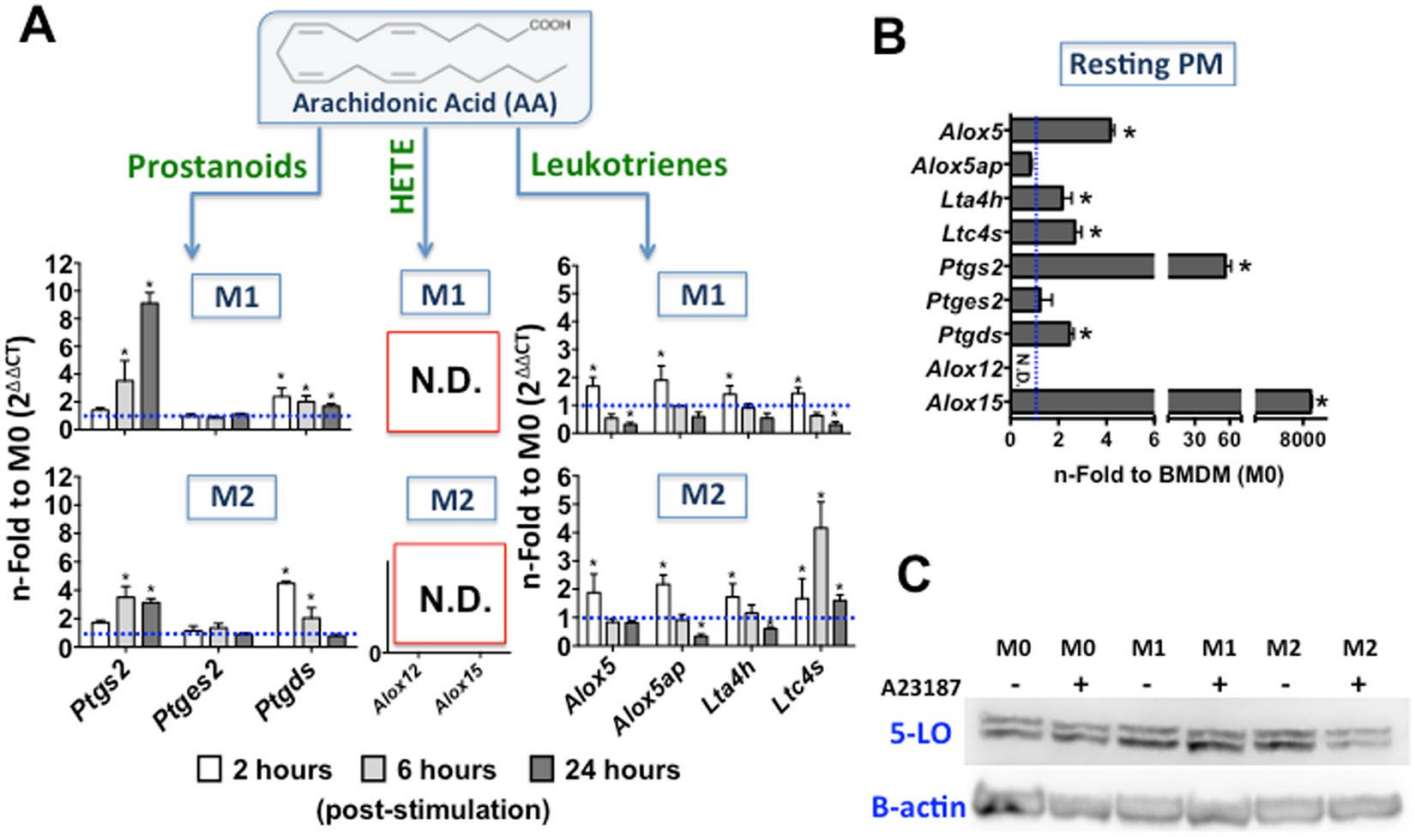
Characterization of macrophage eicosanoid catabolism enzyme mRNA expression and 5-LO protein synthesis. For mRNA expression **(A)** BMDMs were treated with IFN-γ (100 ng/mL) (M1), IL-4 + IL-13 (10 ng/mL) (M2), or only adhered (M0) for 2, 6, and 24 h *in vitro*; or **(B)** resting PMs were used. Total RNA was extracted, synthesized as cDNA, and the relative expression (ΔΔCt) was analysed by qRT-PCR. Transcripts encoding cyclooxygenase and lipoxygenase pathway genes were analysed. The results were normalized to endogenous expression of the internal controls *Actb* and *Gapdh*. The blue dotted lines show the mRNA expression on non-primed BMDMs (M0). The results are presented as the means ± s.e.m. of three independent experiments (n = 3). *p < 0.05 compared to non-primed BMDMs (M0). Qualitative data for **(C)** 5-LO protein synthesis was demonstrated by Western Blot assay using a rabbit-polyclonal anti-5-LO antibody (78 kDa). The membranes were incubated with specific conjugated secondary antibodies (goat anti-rabbit IgG-HRP) and detected with chemiluminescence (ECL) reagent. Representative result from two independent experiments is shown.

Granulocytes, monocytes/macrophages, mast cells, dendritic cells, B-lymphocytes and T cells express 5-LO, whereas platelets, endothelial cells and erythrocytes are 5-LO negative^24, 25^. We therefore evaluated 5-LO mRNA and protein levels in BMDMs. BMDMs were primed with IFN-γ (M1) or IL4 + IL-13 (M2) over 24 h, during which the kinetics of eicosanoid pathway mRNA expression were evaluated. For basal production, BMDMs were adhered in culture for 24 h (M0) with non-primed treatment. The expression of *Alox5* (5-LO), *Alox5ap* (FLAP), and *Lta4h* (LTA4-hydrolase) was up-regulated in macrophages primed to M1 and M2 during the early time (2 h). However, *Ltc4s* (LTC4-synthase) exhibited maximum expression at 6 h post-priming in M2 compared to M0 cells (Fig. 3A). With respect to the COX pathway, we observed an increased expression of *Ptgs2* (COX-2) in the late stage of priming (24 h) for M1 and M2. However, M1 *Ptgs2* expression was greater than that of M2. This mRNA expression profile for COX-2 was in accordance with the prostanoid metabolite production level of these macrophages. Notably, *Ptgds* (PGD-synthase) was up-regulated in M1 and M2 cells after 2 h of priming, whereas non-modulation was observed for *Ptges2* (PGE-synthase 2) (Fig. 3A). These PG-synthase mRNA expression profiles indicate a preference for BMDMs to produce PGD_2_ instead PGE_2_. Finally, the expression of *Alox12* (12-LO) and *Alox15* (15-LO) in polarized BMDMs was not detected (Fig. 3A). To confirm the effect of GM-CSF on eicosanoid enzyme metabolism gene expression, we also performed qRT-PCR (Supplementary Fig. S3B). For LT biosynthesis, we demonstrated up-regulation at an early time point of priming (2 h) for *Alox5* (2.5-fold), *Alox5ap* (3-fold), *Lta4h* (2-fold), and *Ltc4s* (1.5-fold) compared to M0. These results demonstrated potential LT production in primed BMDMs, although the activity of the respective enzymes was not detected.

Post-transcriptional regulatory processes can modulate 5-LO protein expression^26^. In our model, we observed *Alox5* expression in BMDMs; however, the absence of 5-LO metabolite products after stimulation did not provide evidence for 5-LO protein synthesis. Therefore, we polarized BMDMs to M1 and M2, post-stimulated the cells with A23187, and observed the mature 5-LO protein by Western Blot (Fig. 3B). This analysis confirmed the presence of 5-LO protein in non-primed BMDMs (M0), M1 and M2. We also found that this expression was constitutive and independent of A23187 post-stimulation.

For the comparative analysis of mRNA expression and cell eicosanoid metabolism capacity, we characterized the mRNA expression of eicosanoid enzymes pathway genes in resting PMs (Fig. 3C). With respect to LT biosynthesis, we demonstrated a 5-fold expression of *Alox5* compared to that in BMDMs (M0). *Lta4h* and *Ltc4s* expression was up-regulated in PMs to the same level as M1 and M2 BMDMs, when compared with the same reference (M0). However, non-modulation was observed for *Alox5ap* expression. For the COX pathway, *Ptgds* (3-fold) and *Ptgs2* (60-fold compare to M0) expression was up-regulated. However, *Ptges2* expression was not modulated in resting PMs. Moreover, *Alox15* expression was extremely high in PMs compared to M0 whereas *Alox12* expression was not detected in this case.

### AA reacylation by LPATs activity was altered by BMDM polarization

**Figure 4:**
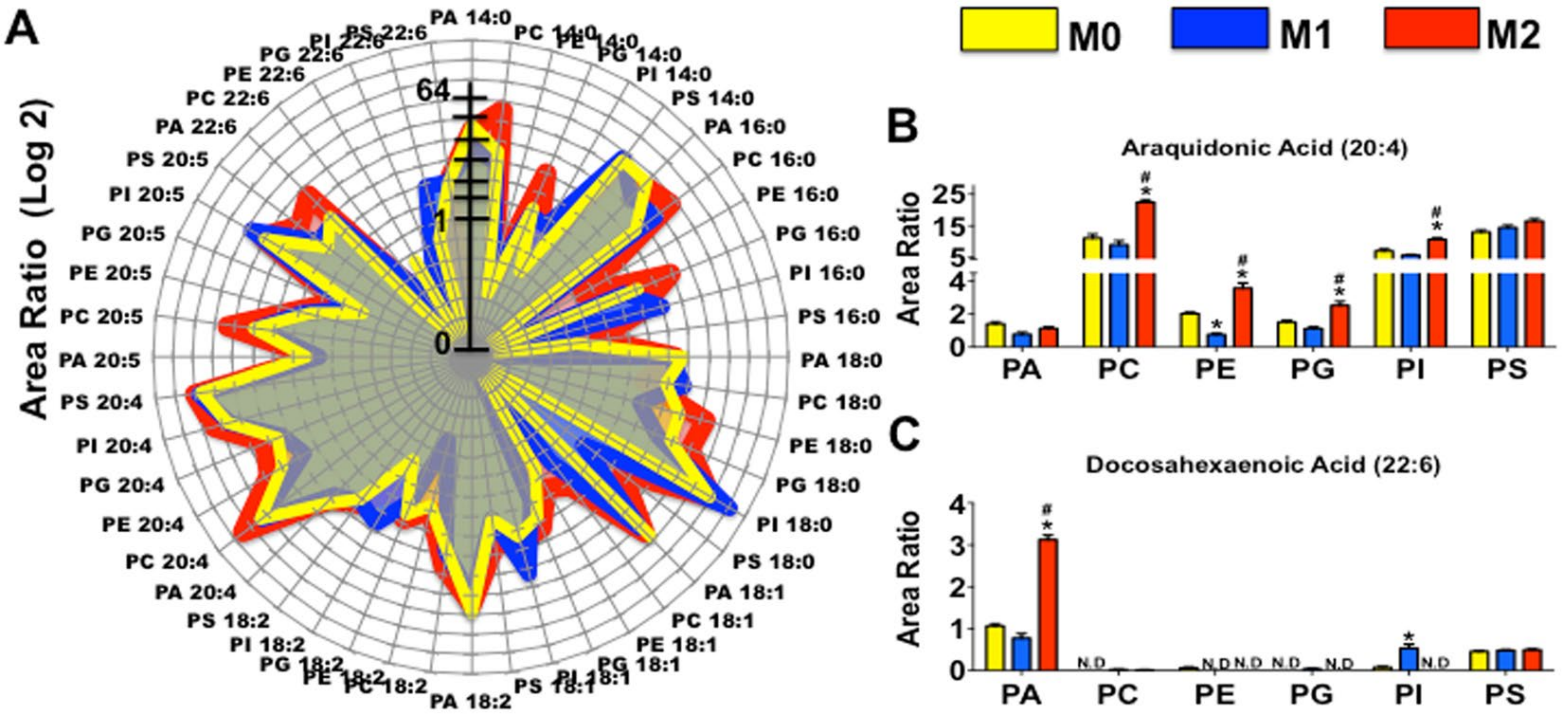
LPAT activity assay by double choice of substrate on polarized BMDMs. Microsomes were used to test BMDM - LPAT activity using a mixture of six lysophospholipids and eight acyl-CoA esters as described in Materials and Methods. The total area ratio of all newly formed phospholipids is expressed in the radar graphic **(A)**. The specific production of AA-anchored **(B)** or DHA-anchored phospholipids **(C)** is represented for polarized BMDMs. The results indicate the area ratio for the incorporation of fatty acid residues in polar groups by internal deuterated standard. Data are representative for BMDMs, 24 h adherent (M0), primed with IFN-γ for 24 h (M1) or with IL-4 + IL-13 for 24 h (M2). All 48 possible products were quantified using HPLC-MS/MS. The Y-axis represents the area ratio (Log2) of newly formed phospholipids. Two independent microsomal preparations were tested in duplicate and the data shown represent the means ± s.e.m. * *p* < 0.05 comparing M1 or M2 to M0; ^#^ *p* < 0.05 for M2 compared to M1.

LPATs are responsible for free fatty acid reacylation to restructure the phospholipids in the cell membrane^27^. Therefore, we next demonstrated the LPAT activity on polarized BMDMs. As no standard dilution curves were used in this case, these results cannot be used to quantitate absolute amounts of the species; however, they are useful to identify potential changes within each phospholipid class between different samples. Figure 4A shows the results of radar graphics analysis of the different possible products formed in the enzymatic reaction. The data represent the relative area (log2) of different species of phospholipids with sn-2 position fatty acids substituents. In adherent M0, the most important points observed were the absence of products PE 16:0, PS 18:0, and PC 18:1, in addition to the diminished levels of PC 16:0, PI 16:0, and PI 18:2 compared to polarized BMDMs. However, M0 demonstrated the most abundant levels of PE 22:6 products. In comparison, stimulation with IFN-γ in M1 revealed another pattern of LPAT activity. Here, the products PI 16:0, PI 18:1, PI 18:2, PI 20:5, and PI 22:6 were higher than those in M0 and M2, whereas decreased production of PE 18:0, PE 18:2, PC 20:4, and PC 20:5 was observed compared to other BMDMs subtypes, along with an absence of PE 16:0 and PE 18:1 species production. In M2 cells (primed with IL-4 and IL-13), the numbers of total identified phospholipid products were vast and the incorporation of different fatty acids exhibited unique patterns in comparison with the other subtypes of BMDMs. M2 exhibited the most production of PC 14:0, PG 14:0, PC 16:0, PE 16:0, PG 16:0, PE 18:0, PS 18:0, PC 18:1, PE 18:1, PE 18:2, PC 20:4, PC 20:5, and PA 22:6, compared to M0 and M1. However, we identified an absence of PC 22:6, PE 22:6, and PG 22:6 products in M2. AA incorporation in phospholipids was greater than DHA in all BMDMs subtypes (Fig. 4B,C). For AA products (Fig. 4B), we observed more formed species for PC, PI, and PS than for PA, PE, and PG. M2 LPAT activity was higher for AA incorporation in PC, PE, PG, and PI, and M1 enzyme activity was significantly lower for PE. Conversely, the DHA (Fig. 4C) incorporated products were prominent only of PA and PS. M2 enzymes activities significantly increased the level of PA 22:6 products, whereas M1 incorporated more PI 22:6 products.

### Negative regulation of 5-LO activity in BMDMs is not affected by the cAMP pathway but is partially susceptible to glutathione peroxidase activity

**Figure 5:**
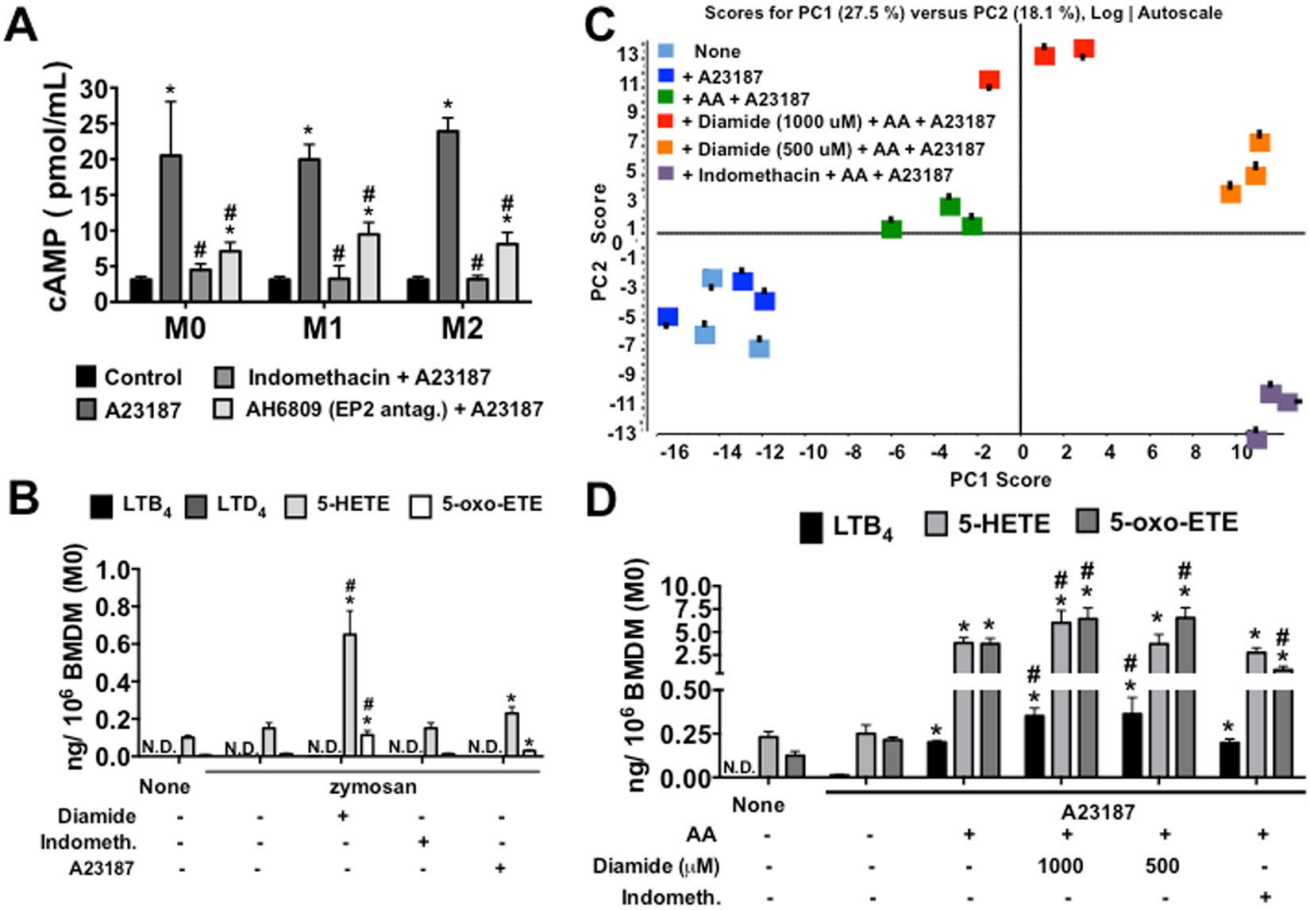
Identification of 5-LO derivative metabolites in BMDMs after exogenous AA stimulation and treatments. BMDMs were treated with IFN-γ (100 ng/mL) (M1), IL-4 + IL-13 (10 ng/mL) (M2) for 24 h, or only adhered (M0). **(A)** cAMP in BMDMs pretreated or not with indomethacin (10 mM) for 30 min, or EP2 antagonist (AH6809, 1 mM); with or without subsequent ionophore A23187 (0.5 μM) stimulation for 9 min (two independent experiments in duplicate, n = 2). Lipids in cell culture supernatants were identified and quantified for BMDMs (M0) pretreated with or without indomethacin (10 mM) for 30 min, or diamide (1000 or 500 μM) for 10 min; with or without subsequent ionophore A23187 (0.5 μM) stimulation for 15 min and/or zymosan (10 μg/mL) for 1.5 h diluted in DMEM. In addition, BMDMs were incubated with or without AA (40 μM) for 10 min before treatment and stimulation. **(B)** HPLC-MS/MS (MRM^HR^) for eicosanoids: LTB_4_, LTD_4_, 5-HETE, and 5-oxo-ETE. **(C)** PCA with Log autoscaling on HPLC-TOF-MS polar lipids. PCA score discrimination of BMDM total polar lipids with or without AA-stimulation and/or ionophore A23187 post-stimulation (n = 3 from each depot, analysed in duplicate). **(D)** HPLC-MS/MS (MRM^HR^) for eicosanoids: LTB_4_, 5-HETE, and 5-oxo-ETE on AA-stimulated BMDMs with or without treatment, and post-stimulation with ionophore A23187 (n = 3, analysed in duplicate). **(A**,**B**, and **D)** The results are presented as the means ± s.e.m. Differences are considered significant when *p* < 0.05, *comparing stimulated-BMDMs versus non-stimulated; ^#^treatment versus non-treated stimulated-BMDMs.

Accessibility and post-translational processing for the regulation of 5-LO activity may also serve as determinants of LT biosynthesis^26^. The most well-known negative regulatory mechanism of 5-LO catalysis is dependent on PKA and indirectly mediated by cAMP release^28^. To assess this factor, we analysed the cAMP production in macrophages culture, as in Supplementary Note. Indeed, high amounts of cAMP were released after A23187-stimulation in all sub-types of BMDMs (Fig. 5A). Furthermore, PGE_2_ signalling through Gs protein-coupled EP2/EP4 receptors, increasing cAMP release^29^. We also determined the pattern of EP receptors expression on macrophages (Supplementary Notes), and the effect of recent prostanoid formation on BMDM cAMP release using a COX-1/COX-2 inhibitor (Indomethacin) (Fig. 5A). Indomethacin inhibited cAMP release from A23187-stimulated BMDMs, independently of polarization. Particularly, PGE_2_-EP2 appeared to have a partial role in BMDM - cAMP production, as demonstrated by EP2 antagonist (AH6809) treatment (Fig. 5A). However, the correlation of PG/cAMP production on the suppressive effect of 5-LO activity was not confirmed in BMDMs stimulated with zymosan (Fig. 5B).

Finally, for 5-LO catalysis, the ferrous iron in the catalytic portion should be oxidized to the ferric form^30^. Additionally, the reduction of lipid hydroperoxide in the cell by glutathione peroxidases may be important for controlling 5-LO activity^31^. To address these issues, we performed experiments using diamide treatment to describe the role of gluthathione peroxidases in BMDM-suppressive 5-LO activity. Figure 5B demonstrates that zymosan stimulation was not able to induce LT production in BMDMs (M0), even when post-stimulated with A23187, but could induce low levels of 5-HETE and 5-oxo-ETE production. However, when zymosan-stimulated M0s were treated with diamide (1000 μM), 5-HETE and 5-oxo-ETE production increased compared to that of cells stimulated with only zymosan, whereas no effect was observed on LT production by diamide treatment of zymosan-stimulated M0.

### Stimulation by AA is important to reversed the 5-LO-dormant activity in BMDMs

Exogenous AA has the capacity to modulate the oxidative metabolism of membrane-derived AA by the 5-LO pathways in ionophore-activated human neutrophils^32^. To assess this capacity in our system, we stimulated BMDMs with AA (40 μM) and post-stimulated with or without A23187. Additionally, we pre-treated BMDMs with diamide or indomethacin prior to the AA + A23187 stimulation. As shown by the PCA scores graphic of the lipid species analysed by untargeted LC-TOF-MS (200–800 *m/z;* Fig. 5C), the AA-stimulated group could be readily discriminated from the non-AA-stimulated group (None or A23187); considering all metabolite ions acquired from the ESI negative ion mode as represented by the corresponding PC Loading. As expected, no extensive differences were observed in PCA between the A23187- and non-stimulated (None) groups. However, the treatment with diamide, at both concentrations of 1000 and 500 μM, reflected distinct PCA data compared to those of AA-stimulated BMDMs, demonstrating a dose-response effect on glutathione peroxidase inhibition. Additionally, indomethacin treatment produced a lipid profile in PCA distinct from that of non-treated AA-stimulated BMDMs. However, the indomethacin pattern data were expressed in the lower quadrant whereas the diamide data were expressed in the top quadrant, highlighting their differences with respect to lipid profiles: indomethacin potentially inhibits the COX-1/COX-2 pathway, whereas diamide was able to increase lipoxygenase and CYP450 products formation.

To gain further insight into the identities of specific lipids that relate to each treatment of BMDMs, and because we focused on 5-LO activity, we restricted the comparison of the BMDM eicosanoids production. Non-production of LTB_4_ was noted upon BMDM stimulation with A23187 (Fig. 5D). However, we observed a basal level of 5-HETE and 5-oxo-ETE production in these groups. Furthermore, we did not observe production of 6-*trans*-LTB_4_, LTC_4_, LTD_4_, 11-*trans*-LTD_4_, or LTE_4_ in any BMDM-treated group. Notably, the AA stimulation reversed the absence of 5-LO activity in BMDMs; after A23187 stimulation, we demonstrated the increased production of LTB_4_, 5-HETE, and 5-oxo-ETE. Similarly, BMDMs treated with diamide (1000 μM) and post-stimulated with AA + A23187 exhibited increased LTB_4_, 5-HETE, and 5-oxo-ETE production in comparison with non-treated AA-stimulated cells. Furthermore, indomethacin treatment did not affect the LTB_4_ and 5-HETE production of AA-stimulated BMDMs but decreased the 5-oxo-ETE production, suggesting that BMDM 5-LO was not susceptible to negative regulation by prostanoids. We also observed a comparable effect of AA stimulation on other lipoxygenases and CYP450 activity (Supplementary Fig. S5). 12-HETE, 15-HETE, and 11,12-EET productions increased in AA-stimulated BMDMs compared with non-stimulated cells whereas diamide treatment increased the production of 12-HETE and 11,12-EET, but not 15-HETE in AA-stimulated BMDMs.

## Discussion

Macrophages are classified as a unique class of cells, albeit with a wide spectrum of phenotypic and genetic variability, among which it has been suggested that Infl-MAs and Res-MAs comprised developmentally and functionally distinct populations^2^. Eicosanoids serve as important molecules for effective regulatory functions in macrophages, acting in both innate immunity and adaptive response^33^. We observed an important bias in the production of eicosanoids when we compared PMs and BMDMs in our conditional model *in vitro*. PMs demonstrated increased PGE_2_ and produced high levels of LTC_4_, LTB_4_, and 5-HETE, whereas BMDMs significantly increased PGD_2_ production and exhibited an absence of 5-LO metabolites after microbial particle stimulation. In this context, we considered that PGE_2_ versus PGD_2_ production and 5-LO-derivative metabolites might act as markers for differentiating those classes of macrophages.

Mouse PMs constitute a well-understood macrophage population in terms of cell biology, development, and inflammatory responses, as well as lipid metabolism for eicosanoids production^19^. However, BMDMs, which are derived using an *in vitro* approach and mimic the process of Infl-MA maturation from bone-marrow cells/monocytes^6^, were expected to exhibit response and functions comparable with those of other primary macrophages. Consistent with this hypothesis, BMDMs are able to produce most soluble inflammatory mediators^22^, mediate inflammasome activation^34^, phagocytosis-associated killing of microorganisms^35^, and secrete mediators of tissue repair, such as VEGF^36^. However, the lack of certain metabolic pathways for eicosanoids production as observed in the current study, influences their response to stimuli and results in substantive deviations of BMDM function from that of PMs. Similarly, another study that utilized stimulation by the TLR4-specific agonist KLA^19^, observed an absence of 5-LO products for TGEMs, BMDMs, and RAW cells; all of which are considered as Infl-MAs and originate from bone marrow. Thus, our results from BMDMs may represent the biology involved in the class of Infl-MAs in mice. In the noted study, the authors suggested that a possible explanation for the impaired 5-LO products on Infl-MAs might be due to down-regulation of the transcript and the lack of Ca^2+^-mobilization by KLA stimulation, which is required for 5-LO activity^19^. Although in the current study we utilized zymosan, a potent Ca^2+^ mobilization agent^37^, as a stimulus.

As BMDMs are immature cells, providing further priming to polarize them toward classical (M1) or alternative (M2) activation is essential. Macrophage polarization is tightly coupled to specific patterns of gene expression^38^. We speculated that the lack of BMDM- 5-LO activity might be reflected by an absence of *Alox5* expression or other post-translational mechanism of 5-LO synthesis. DNA methylation determines whether a cell type can express 5-LO^39^, and naturally occurring mutations in the *Alox5* (5-LO) promoter^40^. Thus, we determined whether the polarization process participated in the gene regulatory expression of the eicosanoid pathway. Although we still did not observe 5-LO product formation in either M1 or M2 polarized cells, we demonstrated that BMDMs expressed mRNA for 5-LO, FLAP, LTA4-hydrolase, and LTC4-synthase, albeit at lower levels compared to those of resting PMs. However, during the polarization of M1 and M2, the expression of *Alox5*, *Alox5ap*, *Lta4h*, and *Ltc4s* increased. Additionally, Western Blot revealed the presence of 5-LO in BMDMs regardless of polarization. Recently, a novel human 5-LO isoform was discovered in primary B and T cells, which was proposed as a novel way to regulated 5-LO activity and subsequently LT production on these cells^41^. However, in the literature there was no evidence of different 5-LO isoform on mice cells.

Another strategy to observe the enhancement of LT production was to prime macrophages with GM-CSF, which is described as a factor for granulocyte priming of LT synthesis subsequent to stimulation^42^. This effect of priming with GM-CSF on increased LT release occurs partially through the enhancement of 5-LO activation, AA release, and increased 5-LO protein levels^43^. We performed priming with IFN-γ (M1) plus GM-CSF, and performed post-stimulation with A23187, a non-specific stimulus that activates Ca^2+^ influx. We identified the eicosanoid profile for GM-CSF-primed M1, which was similar to non-primed M1, as well as absence of 5-LO-derived metabolite formation. Furthermore, we demonstrated increased expression of *Alox5*, *Alox5ap*, *Lta4h*, and *Ltc4s* in GM-CSF-primed M1, indicating that the lack of 5-LO activity in BMDMs was not dependent on gene expression or 5-LO protein synthesis, but rather reflected a potential post-translational regulatory cell pathway that deactivated the 5-LO, or a biochemical mechanism involving 5-LO substrate (AA) release.

In particular, an important regulatory point for LT production is the increase in Ca^2+^ and release of free AA from phospholipids by cPLA2 to effect full 5-LO activation^44^. However, the amount of free AA in cells is not regulated only by cPLA2 but also by a reacylation process to reincorporate free AA into phospholipids by sequential action of acyl-CoA ligase and LPATs^45^, as part of the continuous remodelling of phospholipids through Lands cycle^16^. Consistent with this, other studies have suggested an important regulatory role of AA-reacylation in limiting LT biosynthesis^46^. As demonstrated by their maximal number of total product formation, M2 cells exhibited the highest LPAT activity, followed by M1 as compared to M0. This indicated that the polarization of BMDMs increased the expression of LPATs or their activity. Accordingly, the LPAT activity in M2 reflected a larger number of species of AA- (20:4), and DHA-anchored (22:6) 17:1-phospholipids. In inflammatory cells, AA is generally found to be esterified in the sn-2 position of glycerophospholipids (AA pools), particularly in PC, PE, and PI^47^; we confirmed the enzyme activity underlying formation of these AA pools in BMDMs. Furthermore, in addition to eicosanoids formation, we observed fatty acid release by HPLC-MS/MS. BMDMs exhibited increased AA release, whereas M1 released less AA compared to M0 and M2 when stimulated with LPS or zymosan. This effect may be explained by the enhancement of prostanoid production in M1 cells compared to M0 and M2, leading to the use of free AA as a substrate for COX1/COX2; as well as by the increased LPAT activity on M2 cells, leading to more AA-reacylation on phospholipids. In particular, the release of DHA after stimulation was a prominent characteristic of BMDMs compared to PMs. However, in our lipid analysis, we did not identify metabolites from the DHA substrate. Also, the amount of fatty acids released suggested a normal activity of cPLA2 and Ca^2+^ mobilization in BMDMs.

The regulation of LT metabolism required other biochemical regulatory mechanisms in addition to enzymes and protein transcription^48^. For example, an association of 5-LO with the nucleus has been observed for many cell types^49^ and supports the idea that nuclear membranes constitute the site of LT biosynthesis^50^. Mammalian 5-LOs comprise monomeric enzymes that bind to prosthetic iron^26^. The regulation of 5-LO activity, which is mostly positive, has been shown to involve phosphorylation of Ser-271 dependent on the MAPKAP kinase 2 and 3 (MK2/3) pathway and p38 MAPK; and by ERK2 to phosphorylate 5-LO on Ser-663^26^. Conversely, phosphorylation at Ser-523 by PKA directly suppresses 5-LO catalysis and prevents 5-LO nuclear localization^51^ and this represents the suppressive effects on increased cAMP release, which activates PKA^52^. Notably, polyunsaturated fatty acids such as AA could up-regulate the phosphorylation at Ser-271 and Ser-663, and prevent the cAMP-mediated inhibition of 5-LO translocation and product synthesis in activated neutrophils^53^. We demonstrated a high amount of cAMP release in BMDMs compared to PMs upon stimulated with A23187. The role of PGE_2_ in inducing cAMP release in BMDM was confirmed by the inhibition of PG production via indomethacin treatment or by blocking PGE_2_-ligand receptor interaction with an EP2-antagonist. However, we did not observe LT formation on BMDMs treated with indomethacin or AH6809 and post-stimulated with zymosan or A23187. We supposed that in our model of Infl-MAs (BMDM), the absence of 5-LO activity was not dependent on a negative regulation pathway, but rather on the failure of a positive stimulus to induce LT production. In fact, zymosan acted as a potent inductor of LT production in PMs, whereas LPS diverged to PG formation; and we attested that the zymosan signalling pathway was not disrupted in polarized BMDMs by enhanced on innate-receptor, adaptor molecules and co-receptor expression specific for this ligand.

To further examine the positive regulation, we incubated BMDMs with exogenous AA and post-stimulated with A23187, which resulted in a reversal of the dormant 5-LO activity in BMDMs. Moreover, after AA stimulation we demonstrated production of LTB_4_ and increased formation of 5-HETE and 5-oxo-ETE in BMDMs, but not of other Cys-LTs. The mouse 5-LO was expressed on both transcriptional and translational levels in T cells, and the peripheral blood T lymphocytes were incapable of synthesizing LTs in the absence of exogenous AA^25^. This phenomenon was comparably with our BMDMs results, suggested a common regulatory pathway for LTs production in different myeloid cells. However, another study has shown that exogenous AA stimulation in rat alveolar macrophages preferentially leads to COX products, indicating that AA inhibits agonist-induced LTB_4_ and LTC_4_ synthesis^54^. It should be further noted that after exogenous AA stimulation, diamide treatment, a gluthathione peroxidase inhibitor, enhanced LTB_4_, 5-HETE, and 5-oxo-ETE production in BMDMs. For lipoxygenase catalysis, the ferrous iron of the resting form should be oxidized to the ferric form by lipid hydroperoxides, such as 15-, 12-, or 5-HpETE^55^. Accordingly, glutathione peroxidase added to *in vitro* assays inhibited 5-LO product formation^56^ and no 5-LO activity could be detected in B- lymphocytes unless pre-treatment with diamide was performed^57^. However, indomethacin treatment had no noticeable effect on 5-LO activity, indicating that BMDM- 5-LO expression was not sensitive to a PGE_2_/cAMP/PKA negative regulatory pathway or that AA stimulation prevented this inhibition in our model.

Macrophages are able to respond with appropriate functions in distinct biologic contexts. In turn, eicosanoids play important roles during pathophysiologic processes and must be considered in the development of experimental models, in particular for *in vitro* cell function investigations, such as involving BMDMs. The effects of LTs include leukocyte chemotaxis, release of bactericidal compounds such as defensins and reactive oxygen species, release of cytokines, bronchoconstriction, and vasodilation^58^. Alternatively, we previously proposed an anti-inflammatory activity of LTB_4_ that could abrogate scorpion venom-induced mortality in mice^59^, and suggested that the balance between LTB_4_ and PGE_2_ could be important to control excessive inflammation by inflammasome activation^59^. Moreover, our present results confirm the function of different classes of macrophages on the microenvironment, as Infl-MAs (BMDMs) exhibit high prostanoid production, predicting a pro-inflammatory function. However, we also demonstrated that exogenous AA stimulation could restore the LTB_4_ formation in Infl-MAs. Thus, this study, through comparing the pattern of eicosanoid production of various macrophages and the associated regulatory pathways, allowed the generation of a scenario that may be important for the prediction of the inflammatory response of these cells on the microenvironment, under conditions of physiologic homeostasis or during disease.

## Methods

### Mice

Six–week-old female or male C57BL/6 mice were obtained from The Jackson Laboratory (USA) as well as from the animal facilities of the Faculdade de Ciências Farmacêuticas de Ribeirão Preto, FCFRP/USP. All animal experimental protocols complied with the institutional guidelines on ethics in animal experiments approved by the Animal Care Committee of the Universidade de São Paulo (Permit no. 11.1.468.53.6) and by the Institutional Animal Care and Use Committee at the University of Colorado Denver. All euthanasia was performed under CO_2_/O_2_ excess atmosphere and all efforts were made to minimize suffering.

### Primary macrophage acquisition, culture, and polarization

Murine bone marrow cells were isolated from femurs and cultivated as described previously^22^. The BMDM were plated in microculture plates at a density of 0.5–1 × 10^6^ cells/mL in DMEM supplemented with 10 mM L-glutamine, 100 U/mL penicillin, 100 U/mL streptomycin, and 10% foetal bovine serum (FBS)- DMEM-C and cultured at 37 °C in a humidified atmosphere of 5% CO_2_ for 2 h to allow adherence. Additionally, BMDMs were cultured under the following conditions: (1) in the presence of IFN-γ (100 ng/mL) (R&D Systems, Minneapolis, MN) diluted in DMEM-C for 2 h at 37 °C, to obtain polarization to M1; (2) IFN-γ (100 ng/mL) + GM-CSF (10 ng/mL) (R&D Systems) for 24 h at 37 °C to amplify the stimulatory 5-LO expression; (3) in the presence of IL-4 (10 ng/mL) + IL-13 (10 ng/mL) (R&D Systems) diluted in DMEM for 24 h at 37 °C, to obtain M2; or (3) cultured in DMEM-C for 24 h for adherence at 37 °C in a CO_2_ atmosphere, to obtain phenotype naïve macrophages, designated M0. Resident mouse PMs were obtained from peritoneal lavage as described previously^60^, plated on tissue culture dishes, and kept in a 37 °C incubator for 2 h for adherence prior to stimulation. Macrophages were then washed three times in PBS to remove non-adherent cells and the remaining cells were used for stimulation *in vitro*.

### Stimulation and treatment of primary macrophage cultures *in vitro*

After obtaining the macrophage cultures, the BMDMs or PMs were stimulated or not with LPS (*Escherichia coli*, serotype O111:B4) (0.5 μg/mL) (InvivoGen, San Diego, CA) for 6 h; or with Zymosan (10 μg/mL) (Sigma, St. Louis, MO) for 1.5 h diluted in DMEM at 37 °C in a CO_2_ atmosphere. After macrophage stimulation, lipid mediator production was stopped by the addition of methanol (1:1 *v/v*) direct to the culture cell wells. Additionally experiments, BMDMs were pre-treated with AA (40 μM) (Cayman, Ann Arbor, MI) diluted in DMEM for 10 min. and/or calcium ionophore A23187 (0.5 μM) (Sigma) diluted in Hank’s balanced salt solution (HBSS) supplemented with Ca^2+^/Mg^2+^ (CaCl_2_ −2 mM and MgCl −0.5 mM) for 15 min. at 37 °C. Also, stopped by the addition of methanol. In some experiments, before the stimulus, the BMDMs were treated with specific inhibitors, such as indomethacin (10 μM) (Cayman) for 30 min; AH 6809 (1 μM) (Cayman), an EP2 antagonist, for 30 min; or diamide (1000 or 500 μM) (Sigma) for 10 min. at 37 °C in a CO_2_ atmosphere. All the inhibitors were diluted in serum-free DMEM and the same solution with solvent diluent was used as a control. DMEM was used as a negative control of stimulation. The culture supernatants were harvested and analysed immediately or stored at −20 °C until further use. The absence of stimulus cytotoxicity or proliferative effect was controlled using Resazurin (Alamar Blue®)(Sigma) incorporation.

### Eicosanoid separation and analysis by reverse-phase HPLC coupled to electrospray ionization mass spectrometry (HPLC-MS/MS)

After addition of the internal standards (Cayman) [^2^H_4_]LTB_4_, [^2^H_5_]LTD_4_, [^2^H_8_]5-HETE, [^2^H_4_]PGF_2α_, [^2^H_4_]PGE_2_, [^2^H_4_]PGD_2_, [^2^H_4_]TXB_2_ (2 ng each); [^2^H_5_]LTC_4_, [^2^H_5_]LTE_4_, and [^2^H_4_]6-keto-PGF_1α_ (5 ng each); and [^2^H_8_]AA (10 ng), samples were centrifuged at 800 *g* for 10 min. The supernatant was used to extraction eicosanoids as previously described using a solid phase C18 column^61^. An aliquot of each extracted sample (20 μL) was injected into an HPLC column (Accucore C18 − 50 × 3 mm, 2.6 μm, Thermo Scientific, Waltham, MA) followed by HPLC methodology as previously described^62^. The HPLC system was directly interfaced into the electrospray source of a triple quadrupole mass spectrometer (API 4000, SCIEX, Framingham, MA), wherein MS analysis was performed in the negative ion mode using multiple reaction monitoring (MRM) of specific *m/z* transitions, and quantitation was performed using standard isotope dilution (additional information on Supplementary Methods).

### Eicosanoid pathway gene expression analysis by real-time qRT-PCR

mRNA expression in BMDMs after polarization treatment for 2, 6, and 24 h or in resting PMs was evaluated using a guanidine-based column method to extract the RNA, according to manufacturer protocol (Purelink^TM^, Ambion, Austin, TX). Complimentary DNA (cDNA) was synthesized by reverse transcription of 1.5 µg RNA by using High Quality cDNA Reverse Transcriptase Kits (Applied Bio systems, Foster City, CA). Aliquots of 2 µL total cDNA were amplified by qRT-PCR using Taman^**®**^ in 96-well plates containing primers for *Ptgs2*, *Ptges2*, *Ptgds*, *Ptger1*, *Ptger2*, *Ptger3*, *Ptger4*, *Alox5*, *Alox5ap*, *LTa4h*, *LTC4s*, *Alox12*, *Alos15*, *Ltb4r1*, and *Ltb4r2* in a Step One Plus machine (Applied Bio systems); *Gapdh* and *Actb* were used as reference genes. The results were normalized to the expression levels of the endogenous internal controls *Actb* and *Gapdh*. The 2^−ΔΔCt^ method was used for the analysis of the qRT-PCR data.

### Western blot analysis for 5-LO synthesis

BMDMs were seeded at 3 × 10^6^ per well and polarized to M1, M2, or not (M0) in culture. After macrophage polarization, the BMDMs were post-stimulated or not with A23187. The cells were lysed with RIPA buffer (Sigma) plus a cocktail of protease inhibitors (Complete Protease Inhibitor Cocktail, Roche, Roswell, GA). Proteins from cell lysates were quantified and 50 μg of total protein were resolved by SDS-PAGE (Bolt 4;12% Bis-Tris Plus; Novex-Life). The separated proteins were transferred using a Trans-Blot Turbo (Bio-Rad) onto a 0.45 μm PVDF membrane (Immobilon, Millipore, Billerica, MA). Then the membranes were blocked and incubated with a rabbit-polyclonal anti-5-LO antibody (1:500; 78 kDa; Abcam, Cambridge, UK) followed by incubation with specific horseradish peroxidase-conjugated secondary antibodies (goat anti-rabbit IgG-HRP, Santa Cruz Biotechnologies). The Enhanced Chemiluminescence Luminol (ECL) Reagent (GE Healthcare, Chicago, IL) was used for antibody detection. Images were acquired using a cooled-CCD (Uvitec) imaging system and analysed with Uvitec-Alliance Software (Uvitec, Cambridge, UK).

### LPAT activity assay

Microsomes from BMDMs were prepared as described previously^62^ and protein content was determined by bicinchoninic acid assay (Pierce, Rockford, IL) using BSA as a standard. Then, BMDM microsomes were tested for LPAT activity as described by Martin *et al*.^63^. The assay was performed by analysis of the 48 molecular species potentially generated during the experimental protocol, plus the six deuterated standards.

### Phospholipid extraction and HPLC-MS/MS

Microsome samples were extracted according to the method of Bligh and Dyer^64^. The organic phase was dried under a stream of nitrogen gas and resuspended in 100 μL HPLC solvent mixture from the initial stage of chromatography. Samples were injected into an HPLC system connected to a triple quadrupole MS (API3200, SCIEX) and the normal-phase chromatography was performed using a silica HPLC column (Ascentis, 150 × 2.1 mm, 5 μm, Supelco, Bellefonte, PA). The HPLC methodology was performed as previously described^63^. For the enzymatic assay, MS analysis was performed in the negative-ion mode using MRM. The enzyme activity results was given as the integrated area ratio of different formed phospholipids, corresponding to each MRM signal of the newly synthesized 17:1/X:X, divided by the integrated area of the deuterated internal standard of each target class (additional information on Supplementary Methods).

### Combinatorial screening of untargeted LC-TOF-MS and high-resolution multiple reaction monitoring (MRM^HR^) for eicosanoids

These assays were performed using a Shimadzu HPLC system interfaced with a Hybrid Quadrupole-TOF LC/MS/MS Mass Spectrometer - TripleTOF 5600^+^ (SCIEX Instruments). For the chromatographic separation of eicosanoids, an aliquot of each previously extracted sample (10 μL) was injected into an HPLC Ascentis Express C18 column (100 × 30 mm, 2.7 μm particle size, Supelco) and the HPLC methodology was performed as previously described^65^. The ESI source operated in negative ion mode for TOF-MS and MRM^HR^; scanning was set to an interface heater temperature of 550 °C; ion spray voltage of −4000 eV; curtain gas of 25 psi; ion source gas 1 of 50 psi; and ion source gas 2 of 50 psi. TOF-MS data were acquired with full scan mode from *m/z* 200−700, with a dwell time of 10 ms for individual transitions and declustering potential of −80 eV. The MRM^HR^ declustering potential and collision energy were optimized individually for each analyte, as described in Supplementary Methods. Data were acquired using Analyst® Software (SCIEX) and were reviewed in PeakView® Software (SCIEX). Untargeted data were extracted and aligned using Markerview® workstation (SCIEX) and expressed by principle component analyses (PCA). Quantitation in high-resolution of target eicosanoids was performed using MultiQuant^TM^ Software (SCIEX) and the product ions extracted were cited on Supplementary Methods. Quantitation was performed by calibration curve using dilutions of standards against a deuterated [^2^H_x_] internal standard. The limit of quantification for each target was determined considering the signal to noise ratio (≥3) and linearity of the standard curves with regression coefficients.

### Statistical analysis

The group graphics data were expressed as the means ± s.e.m. Statistical significance was determined using Graph Pad Prism software (Prism, LaJolla, CA) using ANOVA followed by a Bonferroni test. Values of *p* < 0.05 were considered statistically significant. PCA data utilized Log autoscaling, as described previously^66^. Normalization was not used for comparing sample types exhibiting large changes in lipid composition and total amounts.

## Electronic supplementary material

Supplementary Information
