# Supplementary material for: Dormant 5-lipoxygenase in inflammatory macrophages is triggered by exogenous arachidonic acid
Source: Sci Rep. 2017 Sep 8;7:10981. doi: 10.1038/s41598-017-11496-3 (PMC5591212; doi:10.1038/s41598-017-11496-3)
Supplement: Supplementary file 1 — Supplementary Information [file 41598_2017_11496_MOESM1_ESM.pdf]

Supplementary Information

Dormant 5-lipoxygenase in inflammatory macrophages is triggered by exogenous arachidonic acid

Carlos A. Sorgi[1], Simona Zarini[2], Sarah A. Martin[2], Raphael L. Sanchez[1], Rodrigo F. Scandiuzzi[1], Miguel A. Gijón[2], Carlos Guijas[3], Nicolas Flamand[4], Robert C. Murphy[2] and Lucia H. Faccioli[1*]

[1]Departamento de Análises Clínicas, Toxicológicas e Bromatológicas, Faculdade de Ciências Farmacêuticas de Ribeirão Preto, Universidade de São Paulo, Ribeirão Preto, SP 14040-903, Brazil.

[2]Department of Pharmacology, University of Colorado Denver, Aurora, CO 80045, EUA.

[3]Scripps Center for Metabolomics, The Scripps Research Institute, La Jolla, CA 92037, EUA.

[4]Centre de Recherche de l'Institut Universitaire de Cardiologie et de Pneumologie de Québec, Département de Médecine, Faculté de Médecine, Université Laval, Quebec City, Quebec G1V 4G5, Canada.

[*]Corresponding author:

Email: faccioli@fcfrp.usp.br

Supplementary Note

1. Parameters for BMDM polarization description

In Supplementary Fig. S2A-D, we specified the BMDM phenotypes according to soluble mediator production. BMDM (5.0 x 10^5 cells/well) were treated with IFN-γ (M1) or IL-4, IL-13 or IL-4 + IL-13 (M2) for 24 or 48 hours, or not treated (M0), and then were stimulated with LPS (500 ng/mL) for 24h in cell culture. After stimulation with LPS, the supernatant was recovered and used to measure amounts of nitric oxide (NO), TNF-α, IL-10 and IL-6. M1 cells exhibited high levels of released NO and TNF-α production after stimulation with LPS. Priming with IL-4 + IL-13 decreased the NO and TNF-α formation, and increased IL-10 production. In addition, M1 and M2 type macrophages can be distinguished by differential expression of specific genes[1]. We therefore used qRT-PCR to quantify the mRNA levels of M2 phenotype markers after 24 h of priming (Supplementary Fig. S2E-G). We observed that the priming treatment with IL-4 was important to increase the expression of mRNA for arginase-1 and STAT6, whereas in association with IL-13 (IL-4 + IL-13), the mRNA of other M2 markers, such as YM1 and FIZZ1, was up-regulated. We also demonstrated the expression of innate immunity receptors and adaptor molecules in polarized macrophages. In cells primed with IL-4 + IL-13, the mRNA expression for TLR2, TLR1, mannose receptor (MR), dectin-1, MyD88, and TRIF was increased, and non-modulation of *Tlr4* and *Tlr6* mRNA expression was observed. Other important co-receptors for innate immunity, such as CD14, CD18, and CD36, were up-regulated after M2 polarization. Following this phenotype scenario, we adopted the polarization protocol: BMDMs (10^6 cells) were primed with IFN-γ (M1) for 2 h or IL4 + IL-13 (M2) for 24 h, to continuous our experimental approach.

2. Comparative cAMP production and eicosanoids membrane receptors expression in polarized BMDMs

To address the negative regulatory mechanism of 5-LO catalysis dependent on PKA and indirectly mediated by cAMP release[2], we analyzed the cAMP production during macrophage culture. Macrophages were stimulated with or not A23187 in HBSS

Ca^{2+}/Mg^{2+}. The time of maximal cAMP release was found to be 9 min, as determined by a time-response curve (Supplementary Fig. S4A). Notable, the BMDM cAMP release was 10-fold greater than that PMs under the same experimental conditions (Supplementary Fig. S4B). Therefore, we next observed the mRNA expression of PGE_2 receptors (EP1, EP2, EP3, and EP4), and LT receptors (BLT1 and BLT2 for LTB_4; and CysLT1 for LTC_4/ D_4/ E_4) in polarized BMDMs during priming *in vitro* (Supplementary Fig. S4C). *Ptger4* (EP4) mRNA expression was up-regulated in M1 and M2 cells. However, *Ptger2* (EP2) mRNA expression appeared to be up-regulated only in M2s and *Ptger3* (EP3) mRNA expression was up-regulated in M1s. In addition, the mRNA expression of BLT1 and BLT2 was up-regulated in both M1 and M2 cells. To assess the basal membrane expression, we used a flow cytometer assay for EP2 and EP4 expression on BMDMs (M0). As showed in Supplementary Fig. S4D, both EP2 and EP4 were expressed although EP4 expression was significantly lower than EP2 expression in the BMDM membrane.

Supplementary Figures

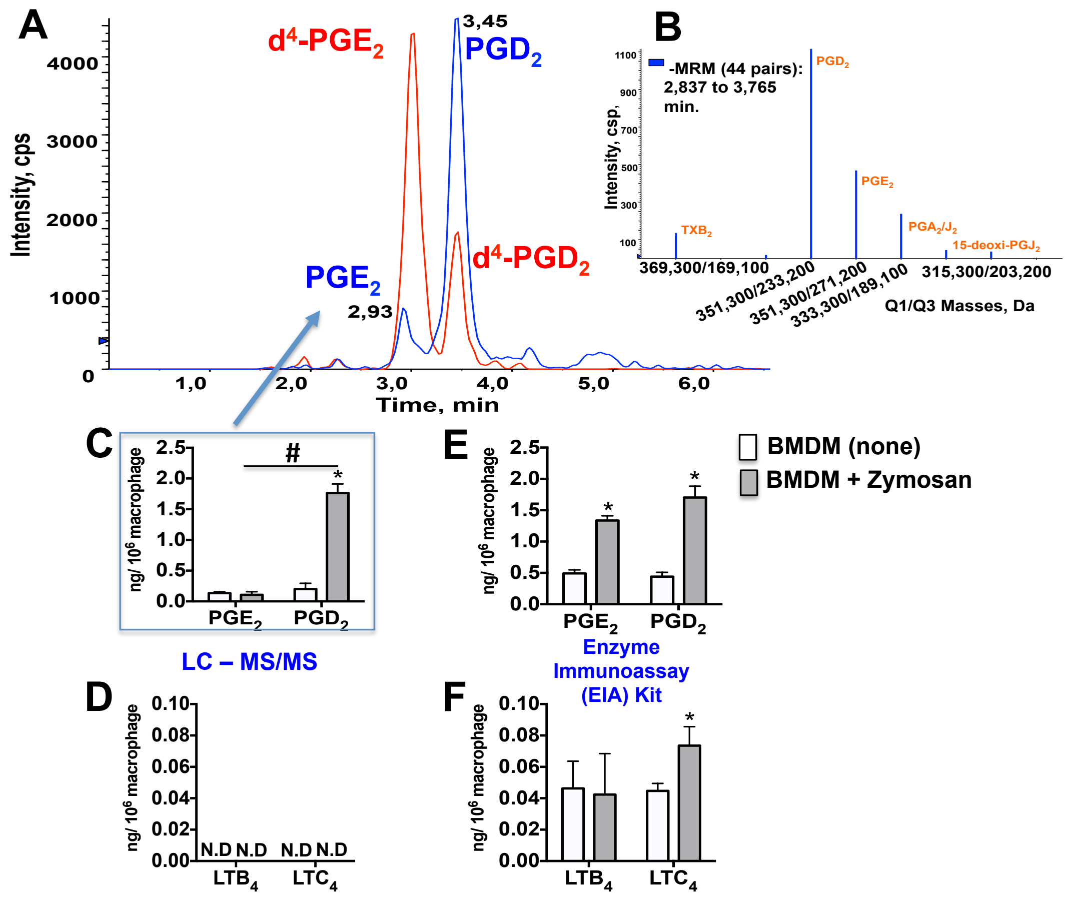

Supplementary Figure S1. Comparative eicosanoid quantification by HPLC-MS/MS and immuno-assay (EIA). BMDMs (1 x 10^6) were treated with IFN-γ (100 ng/mL) (M1) for 2 h. Then, BMDMs were stimulated with zymosan (30 particles/cell) for 1.5 hour. The lipid mediators in cell culture supernatants were identified and quantified. **(A)** Chromatogram of HPLC-MS/MS (MRM mode) for internal standard [$^2H_4$]PGE_2 and [$^2H_4$]PGD_2 (2 ng each) shown as a red line, and the target samples PGD_2 and PGE_2 as blue lines. Representative data of triplicate experiments are shown (n = 3). **(B)** Characteristic Q1/Q3 *m/z* to identify the prostanoids on MRM experiments (one representative experiment). HPLC-MS/MS quantification method for **(C)** PGD_2 and PGE_2 or **(D)** LTB_4 and LTC_4 on BMDMs (M1) stimulated with zymosan. Immuno-assay (EIA) quantification method for **(E)** PGD_2 and PGE_2 or **(F)** LTB_4 and LTC_4 of BMDMs (M1) stimulated with zymosan. Results are expressed as the means ± s.e.m. of three experiments (n = 3). Differences are considered significant when $p < 0.05$, *zymosan stimulated-BMDMs versus non-stimulated and $^{\#}PGD_2$ formation compare to PGE_2.

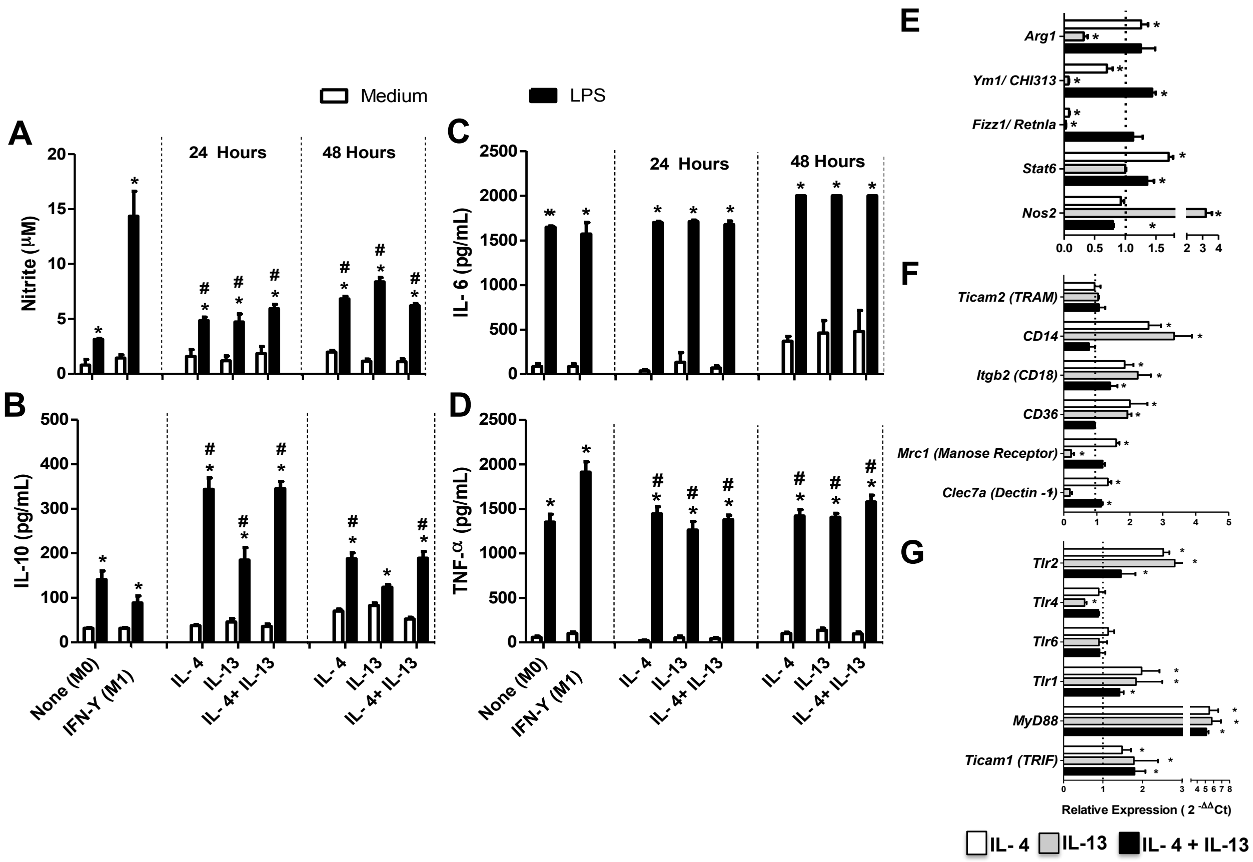

Supplementary Figure S2. Characterization of polarized BMDM phenotypes by inflammatory mediator production and mRNA expression. BMDMs were treated with or without IFN-γ (100 ng/mL) or IL-4 (10 ng/ mL), IL-13 (10 ng/mL) or IL-4 + IL-13 (10 ng/mL) for 24 and 48 h before stimulation with LPS (500 ng/mL). Within the culture supernatant, the amounts of the following components were determined: **(A)** nitrite by Greiss reaction, **(B)** IL-10, **(C)** IL-6, and **(D)** TNF-α by ELISA. For mRNA expression, BMDMs were primed with or not IL-4 (10 ng/mL), IL-13 (10 ng/mL), or IL-4 + IL-13 (10 ng/mL) for 24 h. Total RNA was extracted, synthesized as cDNA, and the relative expression (ΔΔCt) was analysed by qRT-PCR. Transcripts that encode for **(E)** sub-type macrophage phenotypes markers**, (F)** membrane receptors and proteins, and **(G)** TLR-type receptors and adapter proteins were analysed. The results were normalized to endogenous expression of the internal controls *Actb* and *Gapdh*. The dotted lines show the expression of mRNA in non-primed BMDMs (M0). The results are presented as the means ± s.e.m. of three independent experiments (n = 3). **p* < 0.05 compared **(A, B, C, and D)** to control non-stimulated, and **(E, F, and G)** to non-primed BMDMs (M0); #BMDMs primed with IL-4, IL-13, or IL-4 + IL-13 versus M1s (IFN-γ).

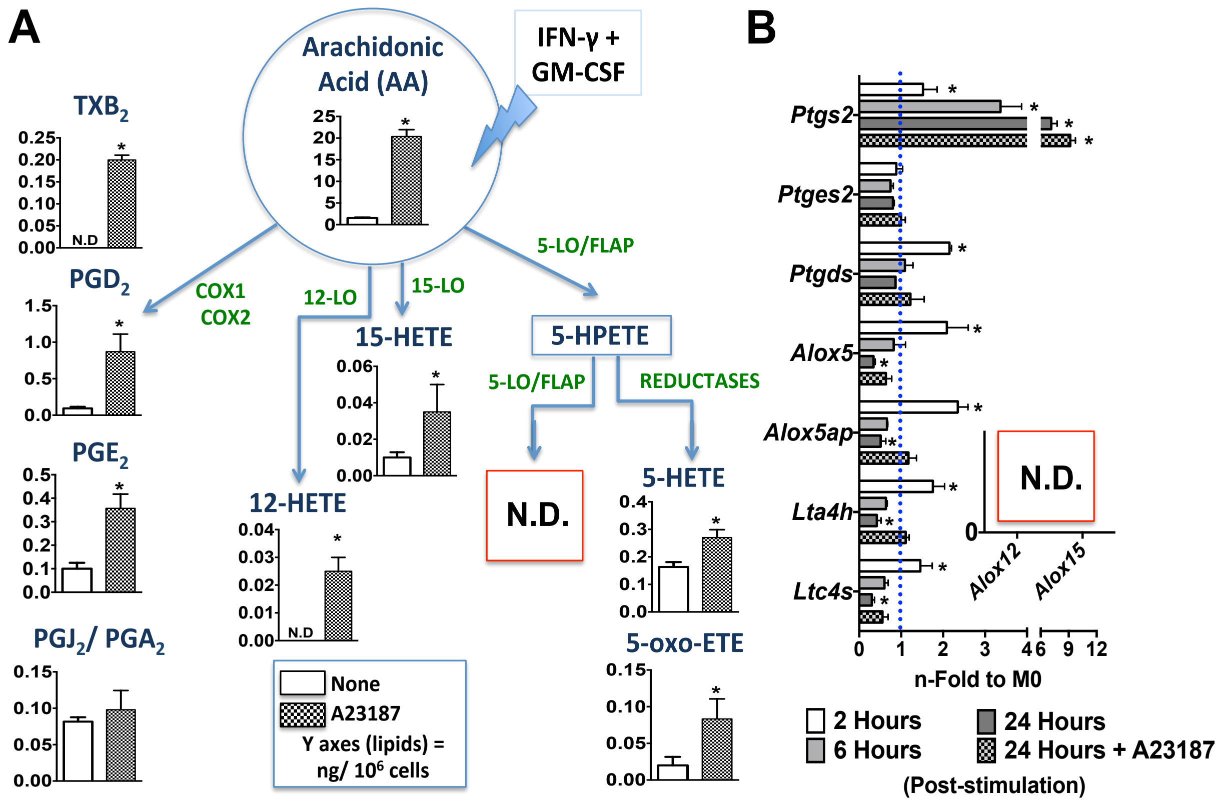

Supplementary Figure S3. Effect of GM-CSF priming on BMDM eicosanoid metabolism pathways. BMDMs were treated with IFN-γ (100 ng/mL) (M1) and GM-CSF (10 ng/mL) until 24 h and post-stimulated with the ionophore A23187 (0.5 μM) for 15 min. **(A)** Lipids in cell culture supernatants were identified and quantified by HPLC-MS/MS (MRM mode) for eicosanoids: TXB_2, PGD_2, PGE_2, PGJ_2/PGA_2, 12-HETE, 15-HETE, 5-HETE, 5-oxo-ETE, and LTs as well as for the release of free AA. The absence of specific eicosanoid production is represented by a red square (N.D.). Results are expressed as the means ± s.e.m. of three experiments (n = 3). Differences are considered significant when $p < 0.05$, *primed BMDMs stimulated with A23187 compared to non-stimulated. **(B)** mRNA expression was determined on GM-CSF-primed BMDMs. Total RNA was extracted, synthesized as cDNA, and the relative expression (ΔΔCt) analysed by qRT-PCR. The results were normalized to endogenous expression of the internal controls *Actb* and *Gapdh*. The blue dotted lines show the mRNA expression of non-primed BMDMs (M0). The results are presented as the means ± s.e.m. of three independent experiments (n = 3). *$p < 0.05$ compared to non-primed BMDMs (M0).

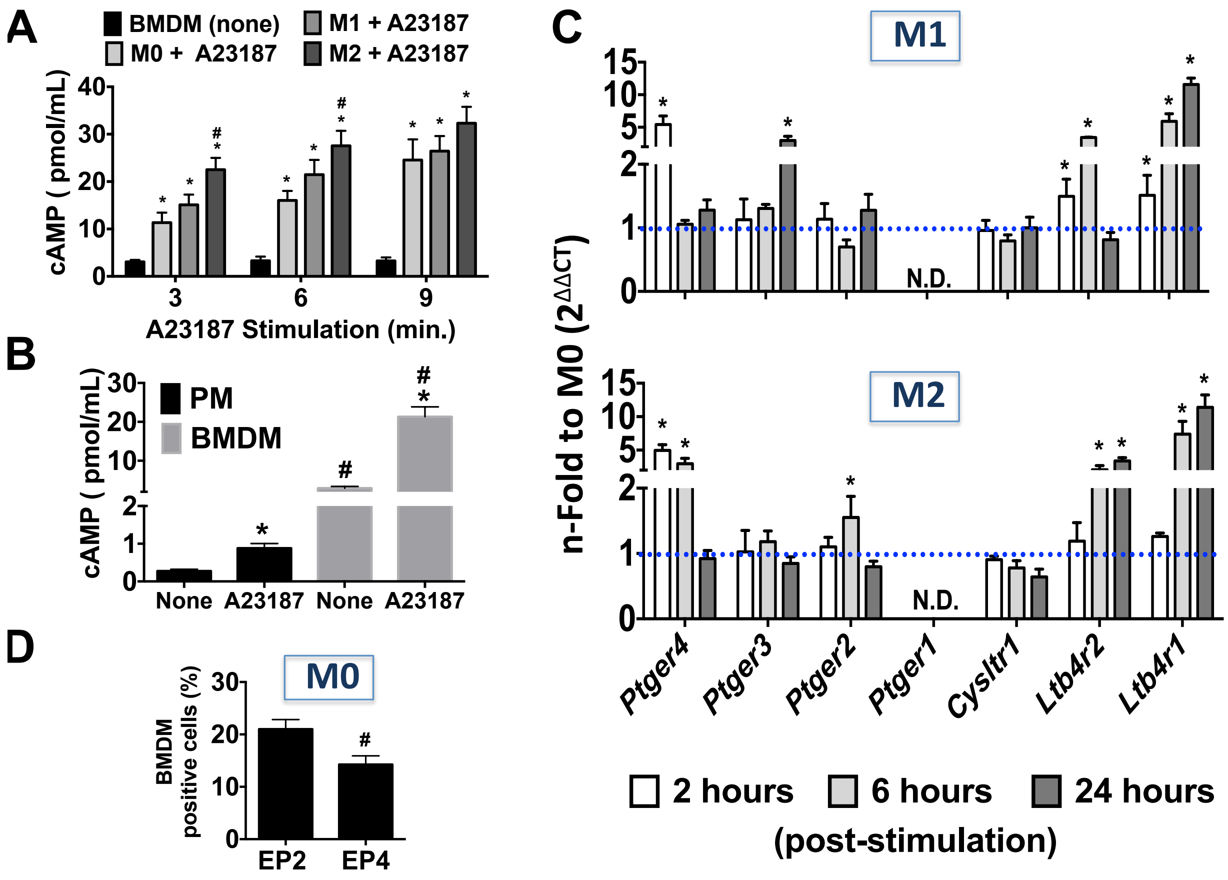

Supplementary Figure S4. Characterization of macrophage cAMP release and eicosanoid receptor expression. BMDMs were treated with IFN-γ (100 ng/mL) (M1), IL-4 + IL-13 (10 ng/mL) (M2), or only adhered (M0) until 24 h *in vitro*. **(A)** cAMP release in BMDMs stimulated or not with ionophore A23187 (0.5 µM) for 3, 6 or 9 min. The results are presented as the means ± s.e.m. of two independent experiments (n = 2). **p* <0.05 compared to non-stimulated BMDMs. #*p* < 0.05 for A23187-stimulated M2 versus A23187-stimulated M0. **(B)** Comparative cAMP release between PM and BMDMs stimulated or not with A23187 (0.5 µM) for 9 min. The results are presented as the means ± s.e.m. of two independent experiments (n = 2). **p* <0.05 compared to non-stimulated macrophage. #*p* < 0.05 for BMDM versus PM. For mRNA expression **(C)** the total RNA was extracted, synthesized as cDNA, and the relative expression (ΔΔCt) analysed by qRT-PCR. Transcripts encoding for PGE_2-receptors and LT-receptors were analysed. The results were normalized to endogenous expression of the internal controls *Actb* and *Gapdh*. The blue dotted lines show the mRNA expression of non-primed BMDMs (M0). The results are presented as the means ± s.e.m. of three independent experiments (n = 3). **p* <0.05 compared to non-primed BMDMs (M0). For membrane expression **(D)** BMDMs (M0) were stained with fluorescence-conjugated antibodies anti-EP2 and anti-EP4, and the relevant

isotype controls. The cells were then analysed by flow cytometry and expressed as the percentage of positive cells for the fluorescent label. We collected and gated 10,000 total events for analysis of live cells as determined by forward and side scatter. The results are presented as the means ± s.e.m. of two independent experiments (n = 2). $^{\#}p < 0.05$ for EP4 compared to EP2 expression on BMDM membranes.

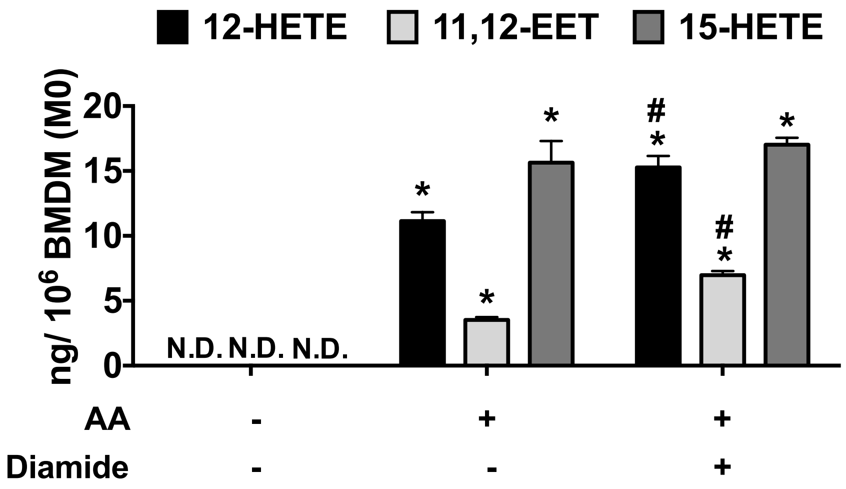

Supplementary Figure S5. Identification of lipoxygenase and CYP450 derivative metabolites in BMDMs after exogenous AA stimulation and treatment. Lipids in cell culture supernatants were identified and quantified for BMDMs (M0) pretreated with or without diamide (1000 μM) for 10 min, followed by ionophore A23187 (0.5 μM) stimulation for 15 min. In addition, BMDMs were incubated with or without AA (40 μM) for 10 min before treatment and stimulation. HPLC-MS/MS (MRM^{HR}) for eicosanoids: 12-HETE, 15-HETE, and 11,12-EET (n = 3, analysed in duplicate). The results are presented as the means ± s.e.m. Differences are considered significant when $p < 0.05$, *comparing AA-stimulated BMDMs versus non-stimulated; #diamide treated versus non-treated stimulated-BMDMs.

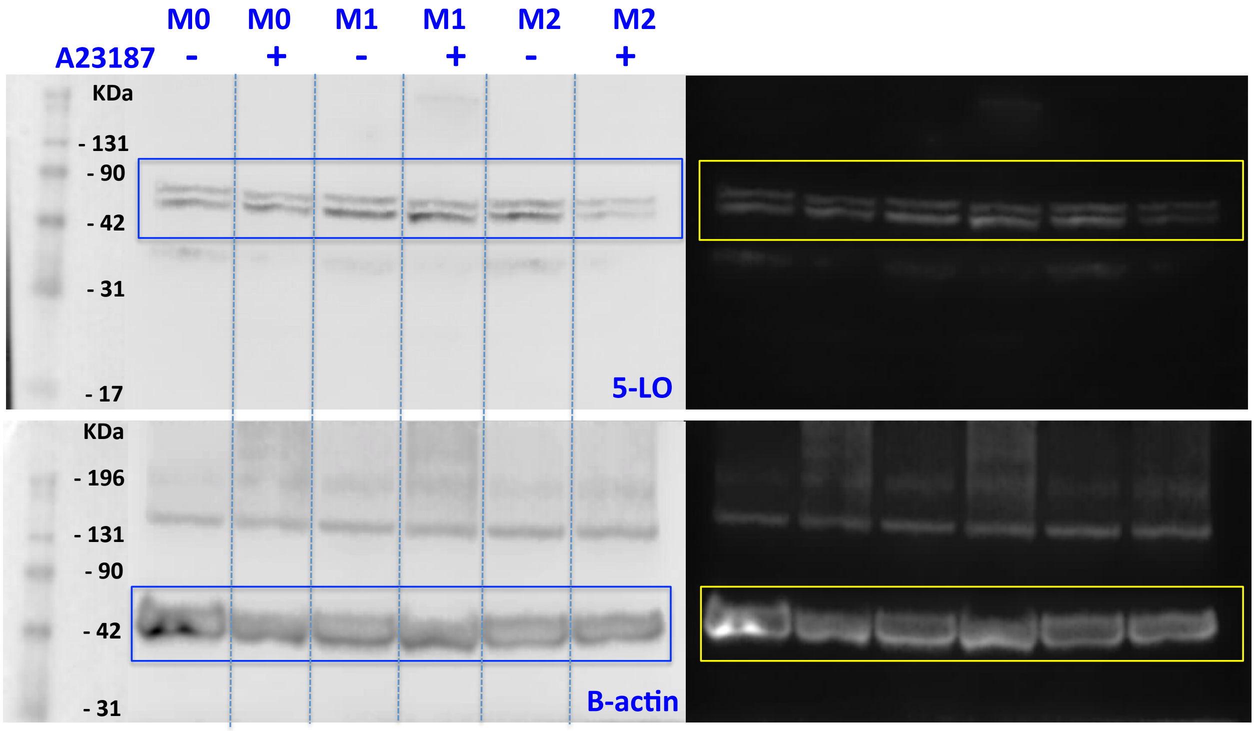

Supplementary Figure S6. Full-length Western blots used in Figure 3C. BMDMs were treated with IFN-γ (100 ng/mL) (M1), IL-4 + IL-13 (10 ng/mL) (M2), or only adhered (M0) for 24 h *in vitro,* and post-stimulated or not with A23187 (0.5 μM) for 15 min. Data shown are expression of total 5-LO protein synthesis (upper panels- 78 kDa), demonstrated by Western blot assay using a rabbit-polyclonal anti-5-LO antibody. The lower panels represented the protein control expression (β-actin - 45 kDa) by Western blot. The membranes were incubated with specific conjugated secondary antibodies (goat anti-rabbit IgG-HRP) and detected with chemiluminescence (ECL) reagent (5 min). The blue-boxed areas are shown in Figure 3C.

Supplementary Methods

Analysis of BMDM phenotype gene and receptor expression by qRT-PCR. Expression of mRNA in BMDMs stimulated with IL-4 (10 ng/mL), IL-13 (10 ng/mL), or IL-4 + IL-13 (10 ng/mL) diluted in DMEM for 24 h at 37°C, to obtain M2, compared to that of phenotype naïve macrophages (M0) cultured in DMEM for 24 h for adherence at 37°C in a CO_2 atmosphere, was evaluated using a custom RT^2 Profiler PCR Array kit (Qiagen, Venlo, The Netherlands). Total RNA from BMDMs was isolated using the RNeasy Mini kit (Qiagen), and the reverse transcription of 3 µg RNA was performed using the RT^2 HT First Strand kit (Qiagen). Each cDNA sample was processed in 96-well plates containing sets of pre-defined gene primers, includes SYBR® Green-optimized primer assays for a specific panel: *Arg1, Chi313, Retnla, Stat6, Nos2, Cd36, Mrc1, Clec7a, Myd88, Tlr1, Tlr6, Tlr4, Tlr2, Cd14, Itgb2, Ticam1*, and *Ticam2,* including *Actb* and *Gapdh* as internal endogenous controls in all sets. Amplification was performed in duplicate in an Eppendorf Mastercycler (Eppendorf, Hamburg, Germany). The $2^{-\Delta\Delta Ct}$ method was used in the analysis of the RT-PCR data.

Cytokine ELISA. Supernatants were harvested and assayed for cytokine content using commercially available ELISA reagents for TNF-α, IL-6, and IL-10 (Duoset R&D Systems, Minneapolis, MN). The detection limit for these cytokines was 7 pg/mL.

Nitrite measurements. Nitrite concentrations in the cell supernatants were measured using the Griess reaction (3% phosphoric acid (Sigma), 1% p-aminobenzene sulphonamide (Sigma), and 1% N-1-napthyl ethylenediamide (Sigma)) as previously described[3].

Flow cytometry analysis. BMDMs were adjusted to a concentration of 5 x 10^5 cells/100 µL and FcγRs were blocked by incubation with unlabelled anti-CD16/CD32 antibodies (BD Biosciences, San Jose, CA) for 40 min at 4°C. Macrophages were stained with the following fluorescence-conjugated antibodies (Abcam): anti-EP2 mAb (PE) and anti-EP4 mAb (APC), and the relevant isotype controls, for 30 min at 4°C in the dark, and washed with PBS plus 2% FBS (v/v). The cells were then analysed using a FACSCanto flow cytometer (BD Biosciences) with FACSDiva software (BD

Biosciences). We collected and gated 10,000 total events for the analysis of live cells as determined by forward and side scatter.

Measurement of intracellular cAMP. For intracellular cAMP measurement, 1 × 10^6 BMDMs (M0, M1, and M2) were stimulated with 1.0 mL A23187 (0.5 μM) diluted in HBSS supplemented with Ca^{2+}/Mg^{2+} for 9 min. at 37°C in 5% CO_2. Cells were also pre-treated with or without Indomethacin or AH 6809 for 30 min. prior to A23187 stimulation at 37°C in a 5% CO_2. Culture supernatants were then aspirated and the cells were lysed by incubation for 10 min with 0.1 M HCl at room temperature, followed by disruption using a cell scraper[4]. Intracellular cAMP was quantified by enzyme-linked immunosorbent assay (ELISA) using an acetylation protocol, according to the manufacturer (Enzo Life Sciences, Farmingdale, NY).

Eicosanoid extraction. Macrophages stimulatory condition medium samples (50% methanol) were centrifuged at 3,000 *rpm* for 10 min. Briefly, the supernatants were diluted with water to a final methanol concentration of less than 15% (*v/v*) and extracted using Strata-X 33u polymeric reverse-phase cartridges (60 mg/1 mL, Phenomenex, CA) preconditioned with 4 mL methanol and 4 mL water. The eluate (in 1 mL methanol) was dried down in a Speed-vacuum concentrator (Eppendorf, USA) and resuspended in a mixture of 40 μL HPLC solvent A (8.3 mM acetic acid buffered to pH 5.7 with ammonium hydroxide) and 20μL solvent B (acetonitrile/methanol, 65/35, *v/v*)[5].

Eicosanoid separation and analysis by reverse-phase HPLC coupled to electrospray ionization mass spectrometry (LC–MS/MS). An aliquot of each lipid sample extracted before (20 μL) was injected into a HPLC column (Accucore C18 - 50 x 3 mm, 2.6 μm, Thermo Scientific, EUA) and eluted at a flow rate of 300 μL/min with a linear gradient of HPLC solvent B, which was increased from 45 % to 75 % in 6.5 min, to 98 % in 1 min, and held at 98 % for a further 6.5 min before re-equilibration at 45 % for 10 min. The HPLC system was directly interfaced into the electrospray source of a triple quadrupole mass spectrometer (API 4000, SCIEX, EUA) where mass spectrometric analysis was performed in the negative ion mode using MRM of the following specific *m/z* transitions (precursor ion → product ion)[6]: 351 → 233, PGD_2; 351 → 271, PGE_2; 369 → 163, 6-keto-$PGF_{1\alpha}$; 353 → 193, $PGF_{2\alpha}$; 333 → 189, PGA_2 and

PGJ_2; 315 → 203, 15-deoxy-PGJ_2; 369 → 169, TXB_2; 351 → 217, LXA_4; 351 → 221, LXB_4; 335 → 195, LTB_4, Δ^6-*trans*-LTB_4 and 5,12-diHETE; 351 → 195, 20-OH-LTB_4; 365 → 195, 20-COOH–LTB_4; 335 → 115, 5,6-diHETEs; 624 → 272, LTC_4; 495 → 177, LTD_4; 438 → 333, LTE_4; 319 → 115, 5-HETE; 317 → 203, 5-oxo-ETE; 319 → 179, 12-HETE; 319 → 219, 15-HETE; 343 → 245 and 343 → 273, 17-OH-DHA; 319 → 191, 5,6-EET; 303 → 205, AA; 327 → 283, DHA; 355 → 237, $[^2H_4]PGD_2$; 355 → 275, $[^2H_4]PGE_2$; 373 → 167, $[^2H_4]$6-keto-$PGF_{1\alpha}$; 357 → 197, $[^2H_4]PGF_{2\alpha}$; 373 → 173, $[^2H_4]TXB_2$; 339 → 197, $[^2H_4]LTB_4$; 629 → 272, $[^2H_5]LTC_4$; 500 → 177, $[^2H_5]LTD_4$; 443 → 338, $[^2H_5]LTE_4$; 327 → 116, $[^2H_8]$5-HETE; and 311 → 267, $[^2H_8]$AA.

Phospholipid extraction and analysis by LC-MS/MS. After addition of the deuterated internal standards $[^2H_{31}]$ 16:0/18:1- PA, $[^2H_{31}]$ 16:0/18:1- PC, $[^2H_{31}]$ 16:0/18:1-PE, $[^2H_{31}]$ 16:0/18:1- PG, $[^2H_{31}]$ 16:0/18:1- PI e $[^2H_{31}]$ 16:0/18:1- PS (25 ng each)(Avanti, EUA), biological samples were extracted according to the method of Bligh and Dyer[7]. Briefly, the organic phase was dried under a stream of nitrogen gas and resuspended in 100 µL of a mixture of 75% HPLC solvent C (hexanes/isopropanol 30:40, *v/v*) and 25% solvent D (5 mM ammonium acetate in hexanes/isopropanol/water 30:40:7, *v/v/v*). Samples were injected into an HPLC system connected to a triple quadrupole mass spectrometer (API3200- SCIEX, USA) and normal-phase chromatography was performed using a silica HPLC column (Ascentis, 150 × 2.1 mm, 5 µm, Supelco, Bellefonte, PA) at a flow rate of 200 µL/min. Solvent D was maintained at 25% for 5 min, increased gradually to 60% in 10 min and then to 95% in 5 min, and was held for 20 min before re-equilibration for 15 min. For the enzymatic assay, mass spectrometric analysis was performed in the negative-ion mode using multiple-reaction monitoring (MRM) of the forty-eight molecular species potentially generated during the experimental assay, plus the six deuterated standards[8]. Results are reported as the ratio between the integrated area of each analyte and the integrated area of the corresponding internal standard for each class. For endogenous phospholipids from BMDM cells, microsomes (10 µg protein), were extracted and analyzed as described above, except that LC–MS/MS analysis was performed using scheduled MRM to detect molecular species containing combinations of common fatty acyl chains[9]. The precursor ions monitored were the molecular ions

[M−H]⁻, except for PC in which case the acetate adducts [M+CH_3COO]⁻ were monitored. Since no standard dilution curves were used in this case, these results cannot be used to quantitate absolute amounts of the analytes, but they are useful to identify potential changes within each phospholipid class between different samples.

MRM^{HR} declustering potential and collision energy for LC-TOF MS/MS. The DP and CE were optimized individually for each analyte (product ions, each 50 ms): LTB_4 – *m/z* 335.2 (q1), DP -15, CE -27 ± 5; Δ^6-*trans*-LTB_4 – *m/z* 335.2 (q1), DP -10, CE -28 ± 5; LTC_4 – *m/z* 624.3 (q1), DP -15, CE -40 ± 5; LTD_4 – *m/z* 495.2 (q1), DP -20, CE -40 ± 5; 11-transLTD_4 – *m/z* 495.2 (q1), DP -20, CE -31 ± 5; LTE_4 – *m/z* 438.2 (q1), DP -10, CE -34 ± 5; 5-HETE – *m/z* 319.2 (q1), DP -20, CE -31 ± 5; 5-oxo-ETE – *m/z* 317.2 (q1), DP -20, CE -30 ± 5; 12-HETE – *m/z* 319.2 (q1), DP -15, CE -28 ± 5; 15-HETE – *m/z* 319.2 (q1), DP -20, CE -31 ± 5; 5,6-EET – *m/z* 319.2 (q1), DP -15, CE -30 ± 5; 11,12–EET – *m/z* 319.2 (q1), DP -10, CE -30 ± 5; [$^2H_4$]LTB_4 – *m/z* 339.5 (q1), DP -15, CE -40 ± 5; [$^2H_7$] 5-oxo-ETE – *m/z* 324.5 (q1), DP -15, CE -34 ± 5; [$^2H_5$]LTD_4 – *m/z* 500.7 (q1), DP -20, CE -26 ± 5; [$^2H_{11}$] 5,6-EET – *m/z* 330.5 (q1), DP -15, CE -37 ± 5; and [$^2H_8$]5-HETE – *m/z* 327.5 (q1), DP -15, CE -40 ± 5.

Product ions for quantitation in high-resolution of target eicosanoids using MultiQuant^{TM} Software. Product ion at ±0.005 Da intervals of mass: LTB_4 and Δ^6-*trans*-LTB_4 – *m/z* 195.1027; LTC_4 – *m/z* 272.0850; LTD_4 and 11-transLTD_4 – 177.0339 plus 143.0412; LTE_4 – *m/z* 235.1526; 5-HETE – *m/z* 115.0350; 5-oxo-ETE – *m/z* 203.1750 plus 129.0520; 12-HETE – *m/z* 179.1040; 15-HETE – *m/z* 219.1360 plus 175.1460; 5,6-EET – *m/z* 191.1770; 11,12–EET – *m/z* 167.1020; [$^2H_4$]LTB_4 – *m/z* 197.1120; [$^2H_7$] 5-oxo-ETE – *m/z* 210.2210; [$^2H_5$]LTD_4 – *m/z* 500.2910 plus 177.0340; [$^2H_{11}$] 5,6-EET – *m/z* 330.3100 plus 202.2500; and [$^2H_8$]5-HETE – *m/z* 265.2550.

Supplementary Reference

1 Zhu, L., Zhao, Q., Yang, T., Ding, W. & Zhao, Y. Cellular metabolism and macrophage functional polarization. *Int Rev Immunol* **34**, 82-100, doi:10.3109/08830185.2014.969421 (2015).

2 Luo, M. *et al.* Protein kinase A inhibits leukotriene synthesis by phosphorylation of 5-lipoxygenase on serine 523. *The Journal of biological chemistry* **279**, 41512-41520, doi:10.1074/jbc.M312568200 (2004).

3 Green, L. C. *et al.* Analysis of nitrate, nitrite, and [15N]nitrate in biological fluids. *Analytical biochemistry* **126**, 131-138 (1982).

4 Peres, C. M. *et al.* Specific leukotriene receptors couple to distinct G proteins to effect stimulation of alveolar macrophage host defense functions. *Journal of immunology* **179**, 5454-5461 (2007).

5 Zarini, S., Gijon, M. A., Ransome, A. E., Murphy, R. C. & Sala, A. Transcellular biosynthesis of cysteinyl leukotrienes in vivo during mouse peritoneal inflammation. *Proceedings of the National Academy of Sciences of the United States of America* **106**, 8296-8301, doi:10.1073/pnas.0903851106 (2009).

6 Zarini, S., Hankin, J. A., Murphy, R. C. & Gijon, M. A. Lysophospholipid acyltransferases and eicosanoid biosynthesis in zebrafish myeloid cells. *Prostaglandins & other lipid mediators* **113-115**, 52-61, doi:10.1016/j.prostaglandins.2014.08.003 (2014).

7 Bligh, E. G. & Dyer, W. J. A rapid method of total lipid extraction and purification. *Can J Biochem Physiol* **37**, 911-917 (1959).

8 Martin, S. A., Gijon, M. A., Voelker, D. R. & Murphy, R. C. Measurement of lysophospholipid acyltransferase activities using substrate competition. *Journal of lipid research* **55**, 782-791, doi:10.1194/jlr.D044636 (2014).

9 Steinhauer, J. *et al.* Drosophila lysophospholipid acyltransferases are specifically required for germ cell development. *Mol Biol Cell* **20**, 5224-5235, doi:10.1091/mbc.E09-05-0382 (2009).
